# K_V_7 channel activation inhibits human and murine myometrium contractility and delays delivery in a mouse model of preterm birth

**DOI:** 10.1038/s43856-026-01861-7

**Published:** 2026-09-03

**Authors:** Yosef T. Mansour, Hiten D. Mistry, Evonne C. Chin-Smith, Mariola K. Zaleska, Deanna C. Sekulich, Jennifer L. Herington, Jeff Reese, Rima Patel, Philip I. Aaronson, Donna M. Slater, Sarah K. England, Iain A. Greenwood, Mark R. Johnson, Paul D. Taylor, Rachel M. Tribe

**Affiliations:** 1 Department of Women and Children’s Health, School of Life Course and Population Sciences, Faculty of Life Sciences and Medicine, King’s College London, London, UK; 2 Division of Public Health & Epidemiology, College of Life Sciences, University of Leicester, Leicester, UK; 3 Department of Pediatrics, Vanderbilt University Medical Centre, Nashville, TN USA; 4 Department of Inflammation Biology, School of Immunobiology and Microbial Science, Faculty of Life Sciences and Medicine, King’s College London, London, UK; 5 Department of Physiology and Pharmacology and Dept. of Obstetrics and Gynecology, Cumming School of Medicine, University of Calgary, Calgary, AB Canada; 6 Center for Reproductive Health Sciences, Department of Obstetrics and Gynecology, WashU Medicine, St. Louis, MO USA; 7 School of Health & Medical Sciences, City St George’s, University of London, London, UK; 8 Faculty of Medicine, Department of Surgery & Cancer, Imperial College London, London, UK

**Keywords:** Reproductive disorders, Medical research

## Abstract

**Background:**

Spontaneous preterm birth, defined as delivery before 37 weeks of gestation, remains a global cause of neonatal illness and death, yet treatments for preterm labor remain limited. We hypothesized that K_V_7 channels in the uterus (myometrium), could be a therapeutic target for preventing or treating preterm labor. The aim was to characterize K_V_7 channel subtypes in human myometrium present after labor onset, and to use a preterm birth mouse model to provide proof-of-principal evidence that activating these channels in vivo can delay preterm delivery.

**Methods:**

This experimental study integrated studies of human myometrial tissue (term and preterm pregnancies, *n* = 159 across experiments), with a RU486-induced preterm birth mouse model (C57BL/6 J, *n* = 6-9 per experimental group), and ex vivo fetal ductus arteriosus preparations (CD-1 mice, *n* = 10 per group). Molecular and protein profiling, isometric tension recordings, and functional pharmacology were used to characterize K_V_7 mediated regulation of human and mouse uterine contractility. In vivo dosing studies in mice assessed the effects of K_V_7 activators (retigabine and ML213) on delivery timing (*n* = 6-9).

**Results:**

We show that K_V_7 channels and ancillary units (KCNE1-5), are expressed in pregnant human myometrium, prior to and after the onset of labor. KCNQ4 and KCNE4 transcripts are also present in myometrium taken at preterm gestations ( ± labor). K_V_7.2-5 activators markedly reduce spontaneous contractions in human and mouse myometrium in vitro and significantly delay preterm birth in vivo with limited impact on fetal ductus arteriosus function.

**Conclusions:**

These findings support K_V_7 activation as a promising approach to suppress uterine contractility and hence delay preterm birth, highlighting a potential new direction for either drug repurposing or therapeutic development.

## Plain language summary

Preterm birth, when a baby is born before 37 weeks of pregnancy, is a major cause of illness and death in newborns. Unfortunately, there are few effective treatments to delay early labor. This study explores whether a group of proteins called K_V_7 channels, found in the muscle of the uterus (womb), could be targeted to prevent preterm labor. We examined human uterine tissue from term and preterm pregnancies and used a mouse model of preterm birth to test drugs that activate K_V_7 channels. We found these channels are present and functional in labor and that activating them reduces uterine contractions and delays preterm birth in mice. This suggests K_V_7 targeting drugs could offer a new approach to preventing preterm labor in the future.

## Abstract (web summary)

Mansour, Mistry et al. perform integrated experiments on pregnant human myometrial tissue and a preterm birth mouse model to examine whether activating uterine K_V_7 channels can delay preterm labor. They report that K_V_7 channels persist in laboring human myometrium and that K_V_7.2-5 activators suppress contractions and delay preterm birth in mice.

## Introduction

Preterm birth (PTB; <37 weeks’ gestation) affects approximately 15 million pregnancies worldwide every year and results in unacceptable levels of neonatal death and morbidity^1^. The resultant social, economic, and healthcare burden for surviving babies is considerable^2^.

A major cause of PTB is the spontaneous onset of preterm labor, typified by inappropriate early onset of uterine contractions and premature cervical shortening. Due to an incomplete understanding of the mechanisms driving preterm labor and the lack of effective prophylactic interventions, many pregnancies still present with threatened preterm labor and require intervention^3–5^. Many will receive, when not contraindicated due to the presence of overt infection, tocolytic therapy to delay labor and corticosteroids to improve neonatal survival and morbidity^5–7^. However, there are few tocolytic therapies available, for example, nifedipine and atosiban (a mixed oxytocin/vasopressin receptor antagonist), but the effectiveness of these agents in terms of delaying birth and impact on neonatal outcomes are limited^5,8^. In addition, the balance of tocolytic versus potential off-target effects, such as cardiovascular changes, is an important consideration. As a result, there is a clinical need to identify new uterine smooth muscle (myometrium) specific targets that are present in laboring tissues to develop more effective tocolytic agents.

Plasma membrane potassium (K^+^) channels are particularly apposite for targeting, as K^+^ channel expression and function underpin uterine quiescence in pregnancy and contribute to the generation of rhythmic contractions in labor^9^. Gene expression of several K_V_ channel subtypes, K_V_1, K_V_2, K_V_4, K_V_7, and K_V_11, and related KCNE ancillary units, has been identified in human and rodent uterine myocytes^9–11^. Of these, Kv7 channels are of particular interest due to their role in generating non-inactivating currents at subthreshold voltages, thereby exerting sustained control over membrane potential and favoring maintenance of myometrial quiescence^12^. We have demonstrated that myometrial K_V_7 channels encoded by KCNQ1-5 genes are expressed robustly throughout mouse gestation. Moreover, activators of K_V_7.2 to K_V_7.5, such as retigabine, inhibited myometrial contractions in mouse and human myometrium (taken at term prior to labor), indicating these channels are not downregulated in late pregnancy^13,14^. K_V_7 channels also play important roles in other excitable tissues, including the cardiovascular and nervous systems, where they regulate vascular tone, cardiac repolarization, and neuronal firing^15–17^. This broader physiological relevance, coupled with our previous publications^13,14^, further highlights the need to define the expression and function of K_V_7 channels within the laboring myometrium.

Considering this, we hypothesize that K_V_7 channels remain active in labor at both term and preterm gestations and represent a promising therapeutic target to delay PTB. The aim of this study, therefore, was to assess K_V_7 channel expression and function in human myometrium and to test whether pharmacological K_V_7 activation delays delivery in a mouse model of preterm birth.

Here, we demonstrate that K_V_7 channel components, including KCNQ and KCNE transcripts and corresponding proteins, are expressed and functional in the pregnant human myometrium before and after labor onset. KCNQ4 and KCNE4 transcripts were also detected in the myometrium from preterm gestations, with and without labor. K_V_7.2-5 activators, retigabine, and ML213 inhibited spontaneous contractions in pregnant human and mouse myometrium in vitro, and retigabine significantly delayed preterm birth in vivo in a non-infectious mouse model. These findings support K_V_7 activation as a promising strategy to suppress uterine contractility and delay preterm labor, highlighting a potential route for drug repurposing and therapeutic development.

## Methods

### Study design

This was an experimental laboratory study using human myometrial tissue, a mouse (C57BL/6 J) model of preterm delivery, and an isolated fetal ductus arteriosus preparation (from CD-1 pregnant mice).

### Human myometrium studies

Human myometrium was obtained, with written informed consent, from 143 pregnant (singletons) pregnancies at term (>37 weeks’ gestation) at the time of elective prior to the onset of labor (i.e., not in the latent phase of labor or experiencing contractions, TNL) or emergency in labor (TAL, after established labor clinically documented) cesarean section, as approved by St Thomas’ Hospital Ethics Committee (and subsequently regulated NRES Committee London-Westminster - (EC00/137), clinical trial number: not applicable. Inclusion and exclusion criteria were similar to those previously published by others^18^. Clinical indications for elective cesarean section were previous cesarean section, breech presentation, and tocophobia. For emergency cesarean section, indications were following labor onset that there were inadequate uterine contractions (dystocia) or compromised fetal status (fetal compromise as indicated by abnormal cardiotocograph). For molecular and isometric tension measurement, all were singleton pregnancies with exclusion criteria of adverse medical and obstetric conditions (including asthma requiring steroids, chronic hypertension requiring medication, lupus/systemic lupus erythematosus, diabetes, and pre-eclampsia) and the use of any medication that had the potential to alter myometrial contractility (e.g., anti-hypertensives and inhaled bronchodilators). Biopsies were obtained from the upper edge of the lower segment incision and placed into ice-cold phosphate-buffered saline. Tissue was rinsed and dissected on ice into small segments, which were either snap-frozen and stored at −80 °C for RNA and protein analysis; formalin-fixed and wax-embedded for immunohistochemical analysis; or kept at 4 °C until used for isometric tension recording. Participant and pregnancy outcome data were retrieved from electronic and hand-held patient notes.

cDNA from an independent, smaller set of human myometrium samples was obtained from D Slater to compare expression in cesarean section samples from preterm deliveries not in labor (PNL, *n* = 4) and after spontaneous onset of preterm labor (PAL, *n* = 5, <37 weeks of gestation pregnancy), with TNL (*n* = 6) and TAL (*n* = 6) controls (Conjoint Health Research Ethics Board REB15-1110).

### Mouse model of preterm birth

All procedures using mouse PTB mouse models and controls were approved by the King’s College London Animal Welfare and Ethical Review Body (AWERB) and conducted per the guidelines and approved protocols in our Home Office project license number 70/7542. Our study complies with Arrive 2 guidelines.

Female C57BL/6 J mice (Charles River) were ordered in, approximately at age 8–12 weeks, and following a minimum of seven days of acclimatization, were mated, and a copulatory plug was visualized one day post copulation (1 dpc); mice were subsequently weighed regularly. Pregnant mice were housed singly in constant temperature rooms (21 °C ± 1 °C) with fixed 12-hour light: dark intervals and ad libitum access to standard chow and water. The progesterone receptor/glucocorticoid receptor antagonist RU486 (mifepristone; Merck Life Science, UK) was used to artificially induce preterm birth as previously published^19^ (Supplementary Fig. 2). On 15 dpc, mice were restrained and a subcutaneous injection containing 150 μg RU486 [dissolved in DMSO (Merck) at a concentration of 1 μg/μl] was administered in the nape of the neck. Control mice were injected with 150 μl of DMSO. All injections were administered at the same time of day, and mice were continuously monitored by video so that the expected outcome measure (hours to delivery post ± K_V_7 activator) and timing of gestation (defined as time to deliver first pup) could be accurately determined. In addition, mice were checked at regular intervals by the research team and animal facility staff, with adherence to protocols and animal wellbeing being overseen by a Home Office veterinary surgeon. General principles of monitoring for weight loss, posture, coat condition, and behavior were employed, with the humane endpoint being a Schedule 1 method cull when concern thresholds were met. In the experiments reported here, no adverse/unexpected outcomes were recorded, and all mice were culled shortly after delivery as per protocol.

For in vitro contraction studies, myometrium was harvested in RU486 mice when they showed signs of labor (preterm in labor, PTAL) and, in parallel, from tissue from control non-laboring pregnant mice at the same gestation (preterm not in labor, PTNL). The outcome measure for molecular analysis was normalized RNA expression, and for isometric tension measurement, it was mean integral tension (MIT).

### In vivo administration of K_V_7 activators in preterm birth mouse model

On 16 dpc, 13 h after RU486 administration, C57BL/6 J mice were administered retigabine (20 mg/kg) or ML213 (20 mg/kg, P.O.) or vehicle (10% (v/v) DMSO in 0.5% hydroxymethylcellulose solution) at 60-minute intervals. Doses were repeated for a maximum of six treatments, or until delivery of the first pup had occurred. The 60-minute interval was based on pharmacokinetic profiles of retigabine administration to female non-pregnant mice (carried out by Quotient Bioresearch, UK), which reported that peak plasma concentrations of retigabine (6930 ng/ml) were achieved approximately 60 min after oral gavage.

### PTB mouse model myometrial tissue collection

For murine myometrium, pregnant mice were sacrificed by cervical dislocation, and the gravid uterus was carefully removed from the peritoneum. Uterine horns were cut along the axis of placental attachment, and the fetus and placenta were excised. Endometrial tissue was gently removed by brushing with a cotton bud. Tissues were either snap-frozen in liquid nitrogen for mRNA extraction or used immediately for isometric tension measurement studies.

### Isometric tension recording

Myometrial contractility was assessed using previously described organ bath methods, with minor modifications^14,20^. In brief, small myometrial strips orientated along planes of fasciculata (human: ~7 x 2 x 2 mm; mouse: ~ 5 x 2 x 2 mm) were mounted and maintained at 37 °C in organ baths (oxygenated, 95% O2, 5% CO2) in physiological salt solution (PSS, pH 7.4 in mM: NaCl 119, KCl 4.7, MgSO4 1.17, NaHCO3 25, KH2PO4 1.18, EDTA 0.025, glucose 6 and CaCl2 2.5). Mouse and human myometrial tissues were subjected to stretch to approximately 1.5-fold their slack length (by applying between 29.34 and 38.25 mN, respectively). After development of regular spontaneous contractile activity with a stable baseline, a control period of contractile activity was recorded prior to the addition of either K_V_7 activators [retigabine, ICA-069673 (Santa Cruz) and ML213 (Tocris)] or inhibitors [XE991 and chromanol 293B (Sigma)] (0.5–20 µM for all compounds). Vehicle control (DMSO) was carried out in parallel. Data in Fig. 5 are from experiments where a single concentration of activator/inhibitor/vehicle was added to an individual myometrial strip; data in Supplementary Fig. 1. shows representative traces where the range of activator/inhibitor concentrations were added cumulatively to a single myometrial strip.

Contractility data were recorded and analyzed using LabChart 6 software (ADInstruments, UK), and MIT for an experimental period, addition of Kv7 activator or vehicle, (the sum of the integrals for each contraction divided by the duration of the period assessed) was expressed as a percentage of the preceding control (baseline) period MIT. Contraction frequency and contraction amplitude (calculated as the difference between baseline tone and the peak force achieved during the contraction) were also assessed.

### Human and mouse myometrium mRNA extraction and real-time qRT-PCR

Frozen myometrial tissue (~100 mg of human or mouse) was homogenized using a Qiagen Tissue Lyser. Total RNA was extracted using the RNeasy mini kit (Qiagen, UK) according to the manufacturer’s instructions. Complementary deoxyribonucleic acid (cDNA) was synthesized using an Omniscript RT Kit (Qiagen, UK). Real-time polymerase chain reaction (PCR) was carried out with the use of SYBR Green chemistry (Bioline) on a RotorGene 6000 (Qiagen, UK) using in- house designed exon-spanning human primers as reported in ref. ^21^ and for mouse as listed in Supplementary Data 1, Table 1, (synthesized by Eurofins, Germany). A pre-PCR cycle was run for 10 min at 95 °C followed by 35 cycles of 95 °C for 15 s, 60 °C for 30 s, and 72 °C for 50 s followed by a final extension at 72 °C for 15 s. Melt curve analysis was performed to confirm the presence of a one-single product. Cycle threshold (CT) values were used for analysis, and abundance data were obtained using quantified cDNA to generate a standard curve. All unknowns fell within the dynamic range of the standard curve. Standards were quantified using densitometry, and tenfold serial dilution from 10^10^ to 10^1^ copies was run in parallel with the samples. Abundance data for the genes of interest were expressed as normalized copy number following normalization using GeNORM (http://medgen.ugent.be/~jvdesomp/genorm), with stably expressed reference genes (glyceraldehyde 3-phosphate dehydrogenase (GADPH). Data for the genes of interest were normalized (GeNorm) to the mRNA copy number of a panel of genes (GAPDH, *β*-actin and *β*−2 microglobulin). All PCR products were sequenced to confirm identity. Expression of each KCNQ and KCNE isoform was measured using cDNA prepared from the same tissue sample within each condition. For analysis of human myometrium KCNQ4 and KCNE4 mRNA expression comparing preterm and term samples, because of limited material, only the housekeeping gene GAPDH was used.

### Immunohistochemistry studies of pregnant human myometrium

Serial sectionparaffin-embeddededded human myometrial tissues were cut (5 µm) and dewaxed from paraffin-embedded tissue blocks (using a Leica RM 2065 microtome, Leica Biosystems, UK) as previously described^21^. Immunohistochemical staining was performed using the Vector Stain Elite ABC kit (Vector Laboratories, UK). The optimal dilution for each antibody was established (see Supplementary Data 1, Tables 2 and 3). A positive control (human midbrain) was used to verify specificity. A negative control was performed for each test section by incubation with goat IgG. All slides were assessed by the same observer, blinded to the labor group. Digital images of 5 randomly selected, high-power (×400 magnification) fields were captured on NIS-Elements F2.20 microscope (Nikon United Kingdom Ltd, UK). Quantifications of KCNQx and KCNEx expression were performed as described previously^22^ using the Positive Pixel Algorithm of Aperio ImageScope software. A visual check was also performed to ensure accurate discrimination of immunolabeled regions.

### SDS-PAGE and Western immunoblotting

Protein samples for Western Blot were prepared by homogenization of human myometrial tissues (TNL samples, *n* = 4; TAL samples, *n* = 4) using a Tissue Lyser II (Qiagen, UK) at 25hZ for 3 min in 20 µl lysis buffer/mg of tissue (10 mM of Hepes-KOH [pH 7], 1 mM of dithiothreitol, 1% nonident-P40, and protease-inhibitor cocktail (COMPLETE tablets; Boehringer-Mannheim Biochemicals, UK) and subsequent centrifugation for 1 min at 12,470 x g to remove any tissue debris. Mouse brain, heart or aorta tissues were used as a positive control for the detection of various K_V_7x/KCNEx proteins and were prepared in the same way as the human myometrium samples. Total protein concentration was calculated using the BCA assay (Pierce, UK) and 30 µg of protein was loaded into each lane of 12% Tris-Glycine precast gels (Generon, UK) and separated using the XCell SureLock™ Mini-Cell system (Invitrogen, UK). Following electrophoresis, proteins were transferred to Immobilion™-P transfer membrane (Millipore) using the XCell SureLock™ Mini-Cell blotting module wet transfer blotting system. 50 mM Tris, 150 mM NaCl, 0.2% (v/v) Tween-20, pH 7.4) containing 5% fat-free milk powder for 1 hour at room temperature. After the transfer, membranes were blocked in TBS-T buffer (50 mM Tris, 150 mM NaCl, 0.2% (v/v) Tween-20, pH 7.4) supplemented with 5% milk (Sigma-Aldrich) for 1 hour at room temperature, followed by 3 h incubation with primary antibody at RT. All K_V_7x and KCNEx antibodies used in this study are listed in Supplementary Data 1, Tables, Table 4. Monoclonal mouse anti-smooth muscle alpha-actin (Sigma, A2547) was used as a smooth muscle protein marker and a loading control. After the first antibody incubation, membranes were washed three times in TBS-T, and HRP-conjugated anti-rabbit, anti-goat, or anti-mouse IgG secondary antibody was added (1:10000 dilution, Santa Cruz). The secondary antibody was incubated for 45 minutes at room temperature, followed by three washes in TBS-T buffer. To detect the antibody, a chemiluminescent reagent (Invitrogen) was added, and the blots were exposed to X-ray film.

### Mouse ductus arteriosus (DA) vessel studies

Animal experiments were conducted in accordance with the National Institutes of Health Animal Care Standards and were approved by the Institutional Animal Care and Use Committee at Vanderbilt University Medical Center. Adult female CD-1 mice were bred by timed mating between 0700 and 1000 h with the presence of a vaginal plug indicating the first day of pregnancy (d1). Pregnant females were anesthetized on the morning of d19 (term) via intraperitoneal injection of 0.4 mL of 1.25% avertin (2,2,2-tribromoethanol in tert-amyl alcohol, Sigma-Aldrich, St. Louis, MO), followed by isoflurane inhalation (Baxter) for fetal anesthesia, and cervical dislocation of the dam. The anesthetized fetuses were removed by cesarean section. Their thoracic cavities were opened via partial dissection and puncture of the diaphragm, and the pups were then submerged in chilled, deoxygenated (95% N2, 5% CO2) modified Krebs (in mM; 109 NaCl, 4.7 KCl, 2.5 CaCl22H2O, 0.9 MgSO4, 1.0 KH2PO4, 11.1 glucose, 34 NaHCO3 (pH 7.3)) (2). Any pups with signs of respiration were excluded from vessel myography.

DA segments representing at least five different litters were freshly isolated from the d19 fetuses for use in myography studies. Each DA was excised, mounted in custom myography chambers (University of Vermont), and allowed to equilibrate for 40 min at 5 mmHg and 37 °C in deoxygenated modified Krebs buffer. The vasoreactivity of each vessel was ascertained using cannulated, pressurized vessel myography as previously described^23–26^. Inverted light microscopes with video capture (IonOptix) were used to continuously record lumen diameter. After the equilibration period, distending pressure was raised by 5 mmHg in 10 min increments to 20 mmHg, followed by two 10-minute exposures to deoxygenated 50 mM KCl with a 20 min wash step of deoxygenated Krebs in between to validate viability and reactivity. After a second 20 min wash step, the chambers were changed from a flow-through system to a 20 mL recirculating volume, and vessels were re-equilibrated for 20 min. At the end of this period, the outcome measure of intraluminal diameter was recorded and used as the baseline value to compare drug-related changes in DA tone. Vessels were exposed to increasing concentrations (10^−8^ to 10^−4 ^M) of either retigabine (Axon Medchem; *n* = 10) or ML213 (Tocris Bioscience; *n* = 10) in 20 min increments, and changes in lumen diameter after each concentration increase were documented and compared.

### Statistics and reproducibility

Formal power calculations determined the sample sizes for KCNQ and KCNE mRNA expression profiles and N numbers for the main experiments. For comparison of KCNQ/KCNE genes within pregnant human myometrium tissue, an n of 21 samples per experimental group was calculated to provide 90% power to detect a ratio of five between genes at the group-wise 5% significance level (alpha = 0.001; S.D. = 0.699), correcting for multiple testing. This was based on data generated from previous experiments^14^. To assess changes in KCNQ/KCNE genes in tissues taken at term prior to labor (TNL) versus term in labor (TAL), an n of 42 (21 per sample group) was calculated to provide 90% power to detect a ratio of five (corresponding to a standardized ratio of 3.5 from previous work) between groups (TNL Vs. TAL) at the group-wise 5% significance level (alpha = 0.0051; SD = 0.544) correcting for multiple testing as above. To determine the effect of K_V_7 modulators on contractile activity of pregnant human myometrium, pilot data have found a consistent standard deviation within treatment groups of 16.6%. With at least *n* = 4 observations in the control and treatment groups, there is 90% power to detect a 33% difference in contractility between the controls and the active treatment. For animal studies (Figs. 6–8), no formal power calculation was performed, but considering the 3 R’s, experimental group numbers were balanced to minimize animal use and informed by our prior ex vivo studies)^14^. In vivo experiments used *n* = 6 animals per treatment group and *n* = 9 for the vehicle control. qPCR analyses and isometric tension measurements used tissues from *n* = 6–8 animals per group. Mice (or tissues) were not randomized to control or treatment groups, and confounders. For in vivo experiments, group allocation depended on the timing of mating success, with control and treatment groups allocated alternatively where possible. For in vitro organ bath studies, control and treated animals, dependent on mating success timings, were run on the same day. There was no systematic allocation of tissues from the control or experimental group to a specific organ bath. Blinding for in vivo studies (and subsequent removal of tissues for in vitro studies) was not employed as a single operator (YM) performed all experiments.

Statistical tests were performed either using SPSS for Windows version 27 or GraphPad PRISM (versions 5 to 11). For expression data, the Kolmogorov-Smirnov test was used to evaluate the normality of the data distribution and summary data are presented as means ± SD or median [interquartile range (IQR)] as appropriate for the data distribution. Between-group comparisons were made using Kruskal-Wallis and post hoc Dunn’s test or Mann-Whitney. The null hypothesis was rejected where *P* < 0.05. Individual figure legends provide specific details.

For the fetal ductus arteriosus (DA) vessel experiments (Supplementary Fig. 3), changes in lumen diameter were analyzed as percent change from the baseline and plotted as mea*n* ± SEM. A comparison of fits determined whether three-parameter non-linear log-fit lines were significantly different between ML213 and retigabine. Two-way analysis of variance followed by a *post hoc* Dunnett’s test was used to determine significant differences in the % change from baseline for each concentration of drug. Two-way analysis of variance followed by a post hoc Sidak’s test was used to determine significant differences between the Emax values of ML213 and retigabine. Sample size was informed by previous work, which indicated that the standard deviation for ex vivo DA constriction in myography studies is ~15%, giving 90% power to detect a ~ 35% change in DA lumen size if *n* = 8 vessels are studied representing different litters. DA tissues used were from *n* = 5 different litters (sex not determined) with *n* = 10 DA tissues for ML213 and *n* = 10 DA tissues for retigabine.

## Results

### K_V_7 channel expression in late-pregnant human myometrium

Therapeutic targeting of uterine K_V_7 channels requires persistence of relevant KCNQ/KCNE transcripts, protein expression, and functional channel activity in the pregnant myometrium after labor onset.

**Fig. 1:**
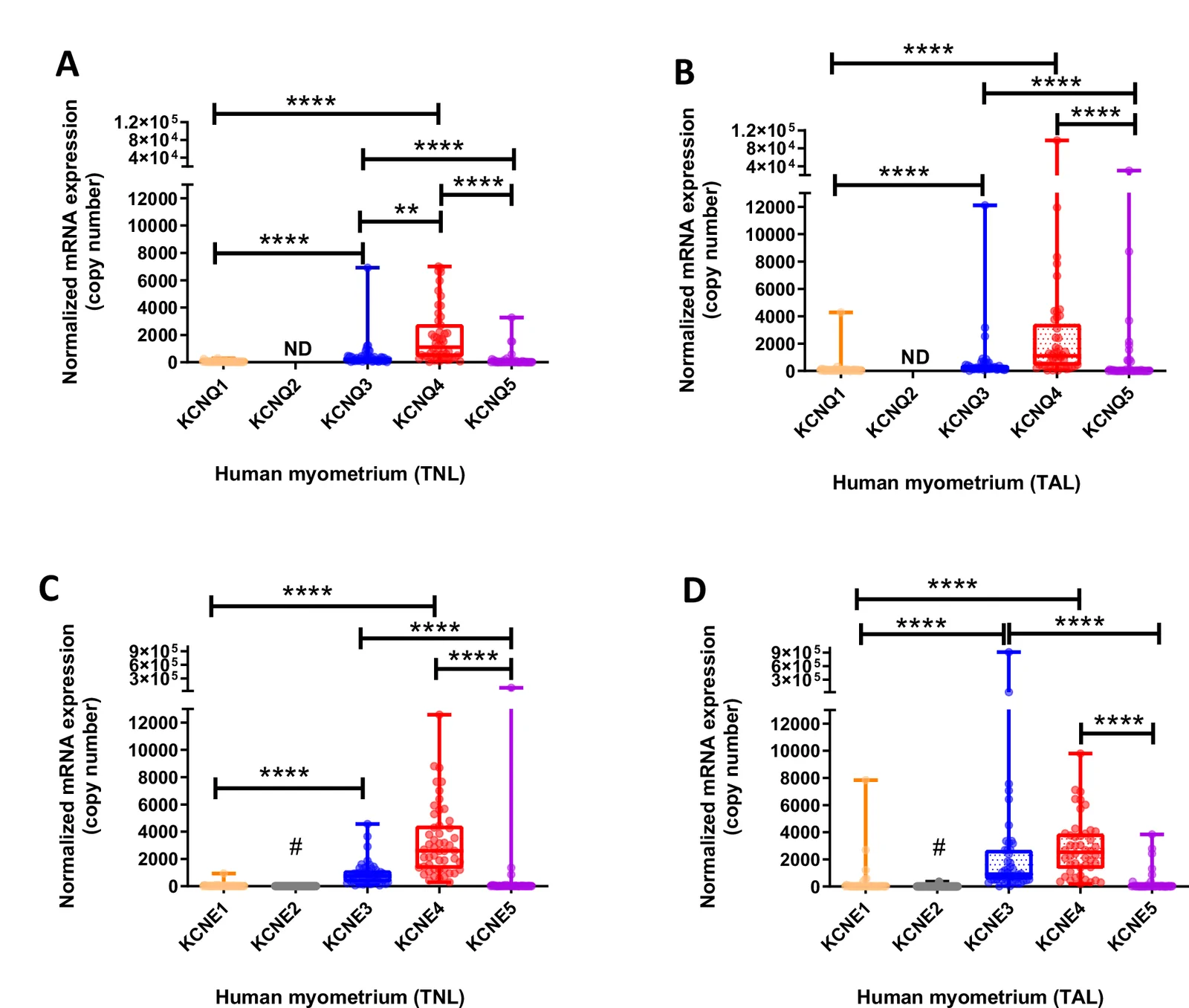
Normalized mRNA expression profile of KCNQ and KCNE genes in pregnant human myometrium samples taken at term prior to labor and during term active labor. Panels show KCNQ expression in term not in labor (TNL; *n* = 51 participants; **A** and term active labor (TAL; *n* = 46 participants; **B** and KCNE expression in TNL (**C**) and TAL (**D**) samples. Expression of each KCNQ and KCNE isoform was measured using cDNA prepared from the same tissue sample within each condition (TNL or TAL). Normalized (GeNorm) mRNA copy number expressed as median and interquartile range and minimum and maximum values. Statistical significance was determined using a one-way ANOVA Kruskal-Wallis and Dunn’s *post hoc* multi-comparison test with adjusted *p* values. ** *p* = 0.0091; **** *p* < 0.001, # *p* ≤ 0.001 KCNE2 compared to all other KCNE genes.

Using myometrium samples taken at the time of elective cesarean section (term not in labor: TNL) (Fig. 1A, *n* = 51 participants), mRNA expression of all KCNQ isoforms except KCNQ2 were detected. Like our previous mouse studies, KCNQ4 was the most highly expressed mRNA compared to KCNQ1,3,5 (*p* < 0.05). The ranked order was KCNQ4»KCNQ3 > KCNQ1 > KCNQ5; Kruskal-Wallis tests confirmed significant differences between the individual KCNQ isoforms (Fig. 1A). Whilst there was a relatively wide spread of KCNQ4 expression values, individual profiles of KCNQ expression were remarkably similar, with KCNQ4 being the predominant isoform in 42 out of 51 tissues (and second highest in the remaining nine samples).

We also assessed KCNQ expression in myometrium samples taken at term in active labor (TAL) (Fig. 1B; *n* = 46 participants). KCNQ expression profiles were broadly similar in TAL and TNL samples, with KCNQ4 remaining the predominant transcript in laboring tissue (KCNQ4 > KCNQ3 > KCNQ1 > KCNQ5). Thus indicating that K_V_7 channel mRNA components are still detectable in labor.

Accessory subunits KCNE1-5 were analyzed in the same TNL and TAL samples (Fig. 1C, D). All KCNE genes were detected in both TNL and TAL samples, with KCNE4 being the most highly expressed isoform in both groups. The expression profile of KCNE genes was the same for both groups (TNL and TAL, KCNE4 > KCNE3 > KCNE5 = KCNE1 > KCNE2). KCNE2 expression was particularly low compared with other KCNE isoforms. KCNE3 showed a slightly lower expression in TNL [median, quartile range KCNE3: TNL: 737.3 (286.0, 1166) *vs* TAL: 915.0 (502.5, 2708).

**Fig. 2:**
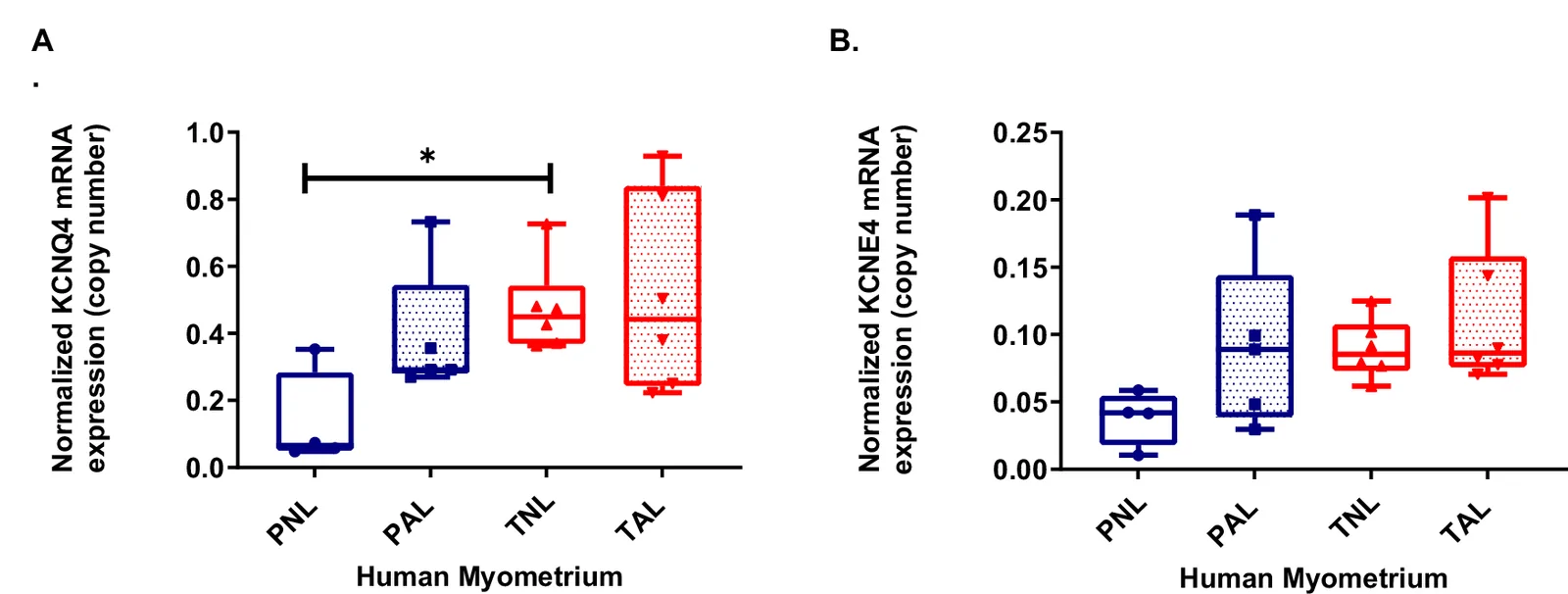
Normalized mRNA expression profile of KCNQ4 and KCNE4 genes in pregnant human myometrium across preterm and term gestations before and after labor onset. Panels show KCNQ4 expression (**A**) and KCNE4 expression (**B**) in myometrium samples taken at preterm gestations (<37 weeks) not in labor (PNL) or in active labor (PAL), and at term gestations before labor onset (TNL) or during active labor (TAL). Samples are independent and additional to data shown in Fig. 1. Normalized (to GAPDH) mRNA copy number expressed as median, interquartile ranges and minimum and maximum values. Statistical significance was determined using a one-way ANOVA Kruskal-Wallis and Dunn’s multi-comparison *post-hoc* test with adjusted *p* values. For A. *p = 0.0494; For B. KCNE4 PNL *versus* TAL, *p* = 0.0525, all other comparison *p* > 0.05. PNL, *N* = 4 participants; PAL, *N* = 5 participants; TNL *n* = 6 participants; TAL *n* = 6 participants.

In a separate experiment, using mRNA from a small set of myometrial tissues (provided by author D Slater), we compared expression from those obtained during cesarean sections for preterm deliveries not in labor (PNL, *n* = 4 participants) and after spontaneous onset of preterm labor (PAL, *n* = 5 participants, <37 weeks of gestation pregnancy), with TNL (*n* = 6) and TAL (*n* = 6) controls. We focused on the most highly expressed KCNQ and KCNE genes identified in Fig. 1. Both KCNQ4 and KCNE4 mRNA (Figs. 2A and 2B, respectively) were detected in preterm tissues. Expression of KCNQ4 was high in PAL as it was in TNL and TAL samples. KCNQ4 expression in the PNL group was lower than that in TNL group (*P* < 0.05). A similar profile of mRNA expression was seen for KCNE4 with a numerical reduction in PNL but this did not reach significant difference in this small study.

Overall, these data show in human myometrium that KCNQ4 tissue expression remains consistent across term and preterm (in active labor) gestations.

### Kv7.1-5 and KCNE1-5 proteins in human myometrium

**Fig. 3:**
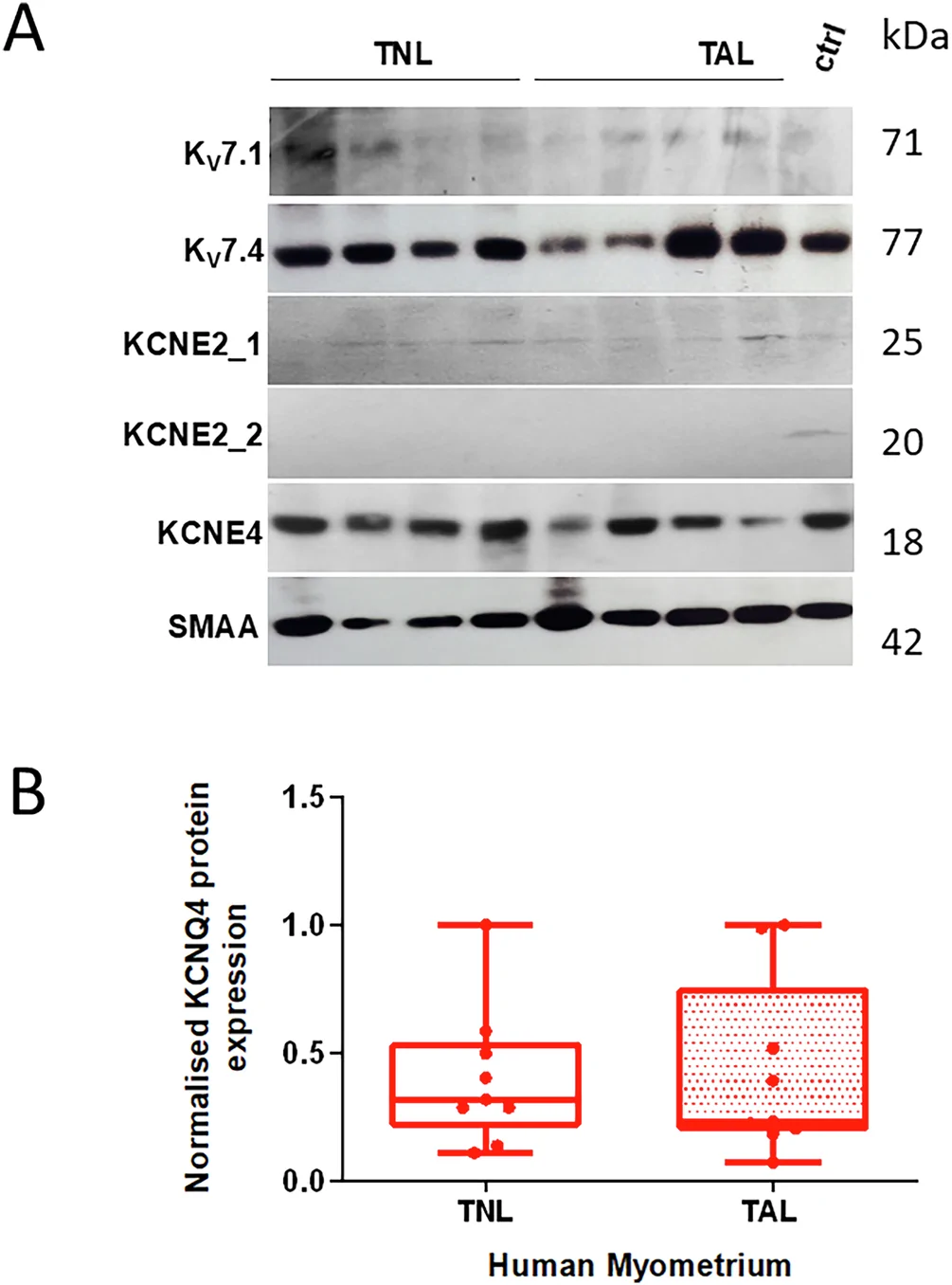
Western blot analysis of K_V_7 and KCNE protein expression in pregnant human myometrium. **A** Representative Western blots showing protein expression in myometrium collected at cesarean section from pregnancies at term not in labor (TNL; *n* = 4 participants) and term active labor (TAL; *n* = 4 participants), with mouse brain, heart, or aorta used as positive controls. Proteins assessed included K_V_7.1 and K_V_7.4, encoded by KCNQ1 and KCNQ4, respectively, KCNE2, KCNE4, and smooth muscle alpha actin (SMAA; loading control). **B** K_V_7.4 expression normalized to SMAA expressed as median, interquartile range and minimum and maximum values. A two tailed Mann-Whitney test showed no significant difference (*p* > 0.05) between K_V_7.4 expression in TNL (*n* = 9) v TAL (*n* = 9) samples.

**Fig. 4:**
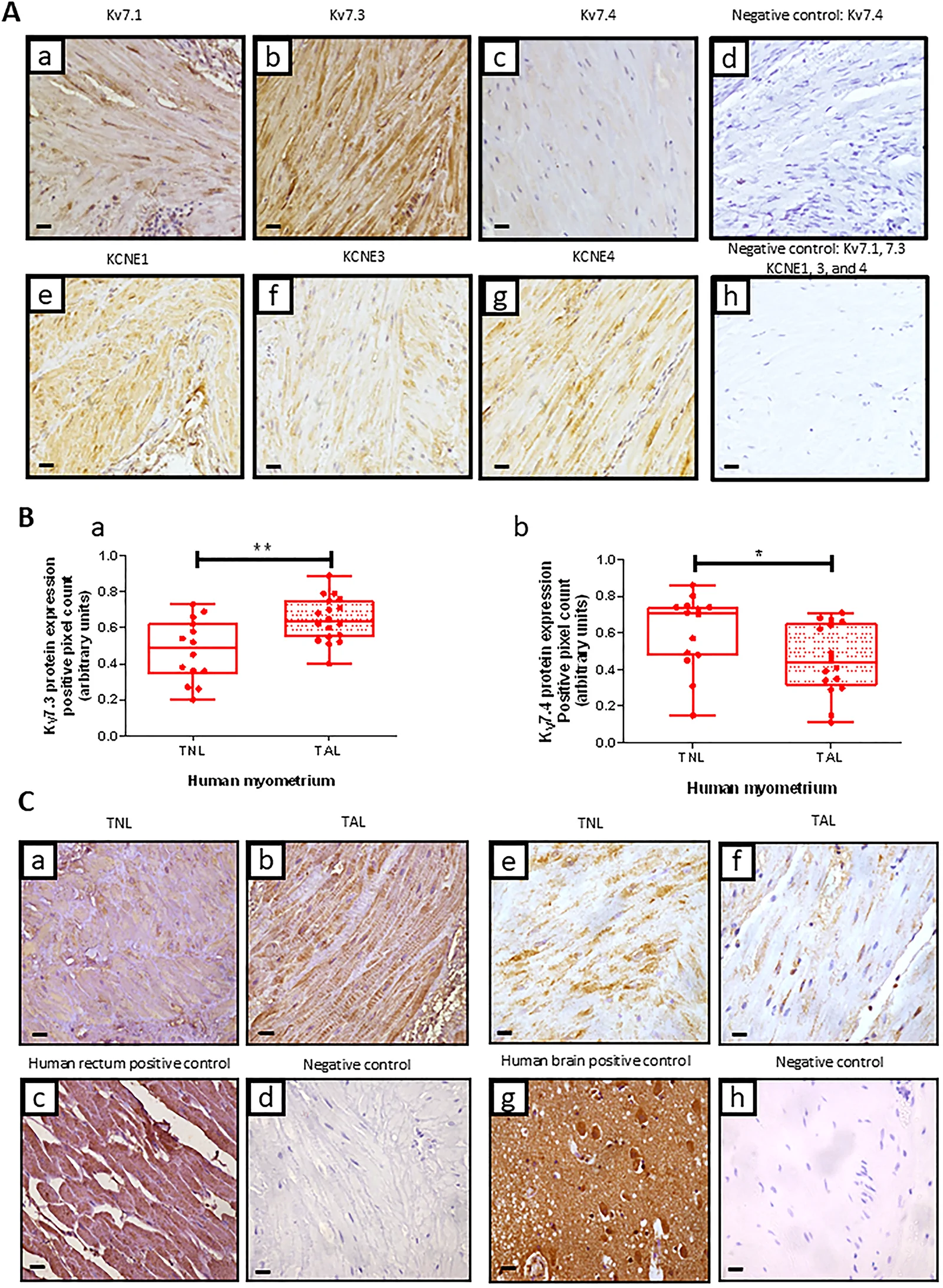
K_V_7 and KCNE isoform immunostaining in pregnant human myometrium samples taken at the time of cesarean section from pregnancies at term not in labor and in labor. **A** Representative immunostaining in term not in labor (TNL) myometrium from individual participants (*n* = 14), showing K_V_7.1 (a), K_V_7.3 (b), K_V_7.4 (c), negative control for K_V_7.4 with no primary antibody (d), KCNE1 (e), KCNE3 (f), KCNE4 (g), and negative control for K_V_7.1, K_V_7.3 and KCNE isoforms with no primary antibody (h). **B** Median positivity for K_V_7.3 and K_V_7.4 staining in TNL versus term active labor (TAL) myometrium. **C** Representative immunostaining for K_V_7.3 in TNL and TAL samples (Ca, Cb) and K_V_7.4 in TNL and TAL samples (Ce, Cf), with positive controls from human rectum and brain (Cc, Cg) and negative controls for K_V_7.3 and K_V_7.4, respectively (Cd, Ch). TNL, *n* = 14 participants; TAL, *n* = 18 for K_V_7.3 and *n* = 16 for K_V_7.4. Box plots show median, interquartile range, and minimum and maximum values. Statistical significance was determined using a two-tailed Mann-Whitney test; *p = 0.0177 and **p = 0.0063. Positive staining, shown in brown, was mainly localized to intracellular areas within smooth muscle cells, with some staining around vessels. Black scale bars represent 100 µm. All photomicrographs were captured at ×400 magnification.

Following the successful detection of KCNQ and KCNE transcripts in human myometrium, we used Western blot (Fig. 3) and immunohistochemistry (Fig. 4) to assess K_V_7 and KCNE isoforms protein expression in human myometrium using commercially available antibodies Kv7.1-5/KCNE1-5 proteins^14,27–33^. Figure 3A shows Western blot detection of Kv7.1, Kv7.4, KCNE2, and KCNE4 proteins. Of the two KCNE2 antibodies, only one antibody (APC-054, Alomone; epitope – 88-107 aa, rat origin) detected a faint protein band (Fig. 3A). APC-054 appears to detect the fully glycosylated protein, whereas the second antibody (sc-25703, Santa Cruz, epitope – 1–70 aa, human origin) detects the semi-mature monoglycosylated protein. Focusing on K_V_7.4, we quantified expression in whole tissue lysates from human myometrium in TNL and TAL samples. Figure 3B demonstrates that protein expression was similar in both groups.

Immunohistochemistry provided further confirmation of the presence and localization of K_V_7.1, K_V_7.3, K_V_7.4, KCNE1, KCNE3 and KCNE4 proteins in pregnant human myometrial smooth muscle (Fig. 4). All were expressed in smooth muscle cells and widely distributed across cells. Semi-quantification of the staining indicated that K_V_7.3 was slightly increased in TAL v TNL samples and K_V_7.4 reduced (Fig. 4B, C). Both proteins were detected in laboring samples, supporting continued expression after labor onset.

### Pharmacological evidence for functional K_V_7 channels in human myometrium

**Fig. 5:**
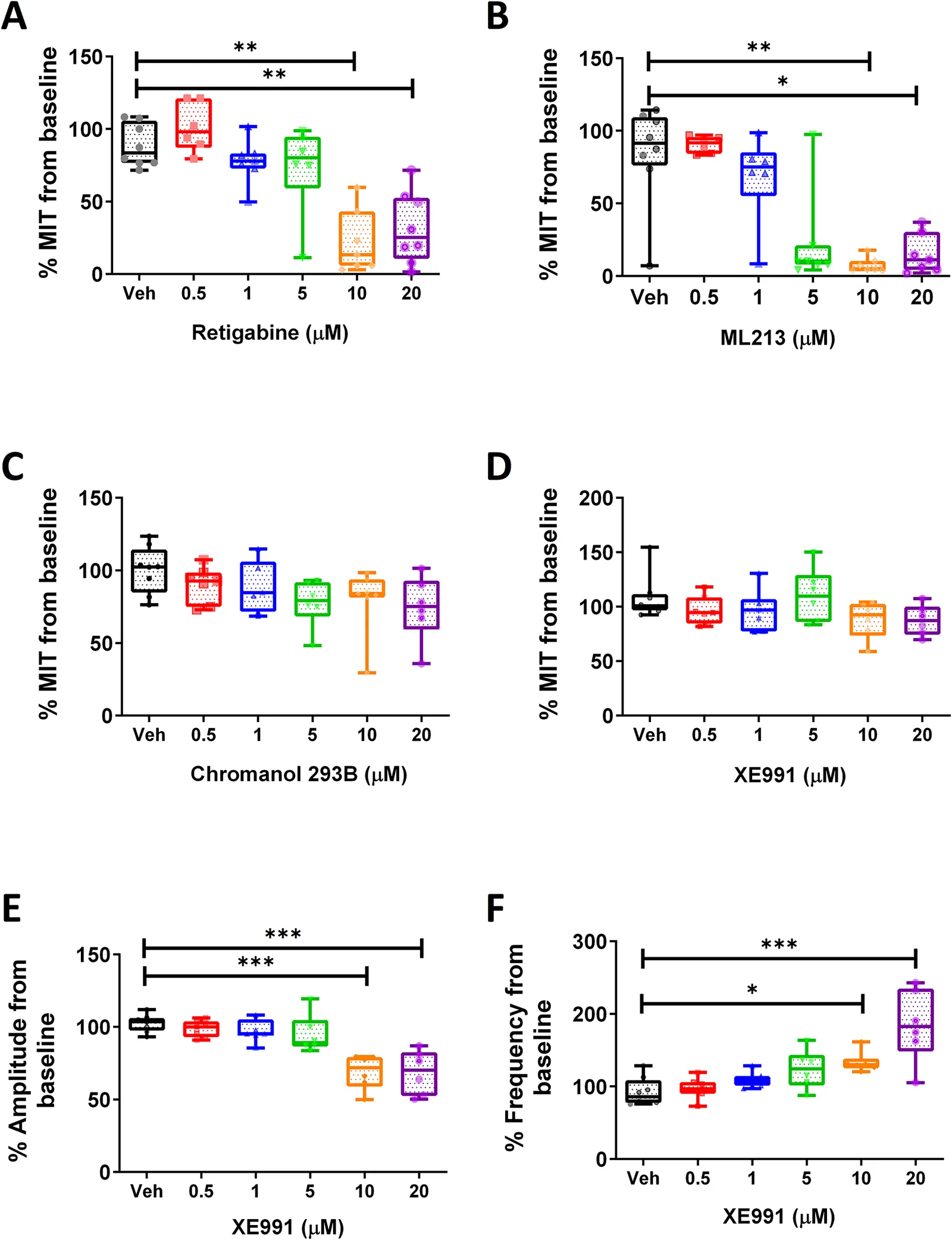
Effects of K_V_7 modulators on spontaneous contractility of human term not in labor myometrium ex vivo. Single concentrations of K_V_7 modulators (0.5–20 µM) were added to individual spontaneously contracting myometrial tissue strips from term not in labor (TNL) pregnancies in organ baths. **A** Retigabine and **B** ML213 effects on mean integral tension (MIT) over 60 min, compared with vehicle control (10 µL DMSO). **C** Effect of the K_V_7.1 inhibitor chromanol 293B on MIT. **D–F** Effects of the pan-K_V_7 inhibitor XE991 on MIT, contraction amplitude and contraction frequency, respectively, compared with vehicle control. Data are expressed as percentage MIT, amplitude or frequency relative to the preceding baseline period before addition of the K_V_7 modulator, and are shown as median, interquartile range, and minimum and maximum values. Statistical significance was determined using the Kruskal-Wallis test with Dunn’s post hoc multiple-comparison test and adjusted *P* values for a priori comparisons between vehicle and individual concentrations. *p < 0.05; **p < 0.01. Each experimental condition, including vehicle control, was tested using myometrial tissue strips from *n* = 6–8 participants; see Supplementary Data 2 for specific n per group. **A** Retigabine MIT: vehicle versus 10 µM, *P* = 0.0028; vehicle versus 20 µM, *P* = 0.0069. **B** ML213 MIT: vehicle versus 10 µM, *P* = 0.0019; vehicle versus 20 µM, *p* = 0.0199. **C** Chromanol 293B MIT: Kruskal-Wallis test not significant overall. **D** XE991 MIT: all comparisons non-significant. **E** XE991 amplitude: vehicle versus 10 µM, *p* = 0.0006; vehicle versus 20 µM, *P* = 0.0008. **F** XE991 frequency: vehicle versus 10 µM, *p* = 0.0124; vehicle versus 20 µM, *p* = 0.0007.

Evidence for functional K_V_7.2-5 channels was gained through a series of in vitro protocols which assessed the impact of pharmacological K_V_7 activators and inhibitors on TNL human myometrium contractility (Fig. 5 and with representative cumulative traces shown in Supplementary Fig. 1).

Application of the K_V_7.2-5 activator retigabine (10 and 20 µM) to spontaneously contracting pregnant human myometrium (TNL) resulted in a reduction in mean integral tension (MIT) compared to vehicle time controls (dimethylsulfoxide, DMSO; Fig. 5A) providing evidence of functional K_V_7.2-5 channels in these tissues. ML213, a K_V_7.2/K_V_7.4 and K_V_7.5 activator caused a reduction in MIT at doses 10 and 20 µM, indicating it is an effective uterine relaxant (Fig. 5B).

There was no significant effect of chromanol 293B, an inhibitor of K_V_7.1/KCNE1 on spontaneously contracting myometrium (Fig. 5C and Supplementary Fig. 1.) consistent with our previous mouse myometrium data^13^.

In contrast, the pan K_V_7 inhibitor XE991, at 10 and 20 µM had no significant effect on mean integral tension but significantly enhanced contractile frequency (Fig. 5D, E) and decreased contraction amplitude (Fig. 5F) compared to vehicle control in TNL pregnant human myometrium. This is consistent with XE991 causing a small depolarization of the resting membrane potential due to inhibition of K_V_7 channels.

Together, these data support a functional contribution of K_V_7.2–5 channels to term pregnant human myometrial contractility, with responses to ML213 suggesting a prominent role for KV7.4-containing channels. K_V_7.1 has minimal functional contribution to contraction and an explanation for this may be that K_V_7.1 is forming channels with KCNE4 (which is the most abundant isoform) in myometrium; this combination is reported to suppress K_V_7.1 currents in model systems^34,35^.

### K_V_7 channels in myometrium from a mouse model of preterm birth

**Fig. 6:**
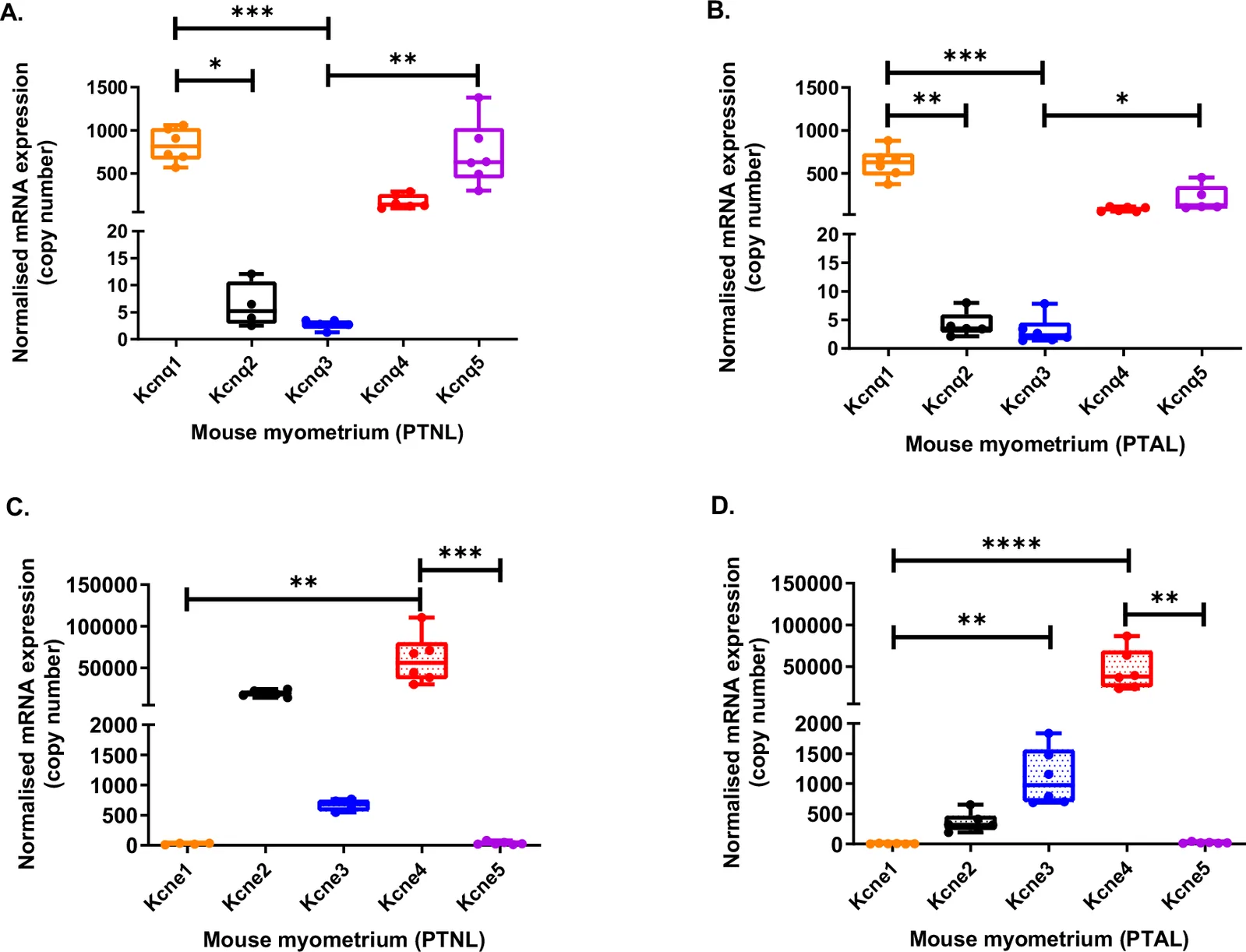
Normalized mRNA expression profiles of Kcnq and Kcne genes in pregnant mouse myometrium from a RU486-induced preterm birth model. Myometrium was collected on day 16 post conception from preterm not in labor mice (PTNL treated with DMSO; *n* = 6) and preterm active labor mice after RU486 treatment (PTAL; *n* = 6). Panels show normalized GeNorm mRNA copy number for Kcnq1–5 in PTNL (**A**) and PTAL (**B**) myometrium, and Kcne1-5 in PTNL (**C**) and PTAL (**D**) myometrium. Data are expressed as median, interquartile range, and minimum and maximum values. Statistical significance was determined using the Kruskal-Wallis test with Dunn’s post hoc multiple-comparison test. Adjusted P values are shown for the indicated comparisons: A, *p = 0.0179, **p = 0.0051, ***p = 0.0010; B, *p = 0.0334, **p = 0.0040, ***p = 0.0001; C, **p = 0.0018 and ***p = 0.0006; D, **P = 0.0045 for Kcne1 versus Kcne3 and *P* = 0.0035 for Kcne4 versus Kcne5.

**Fig. 7:**
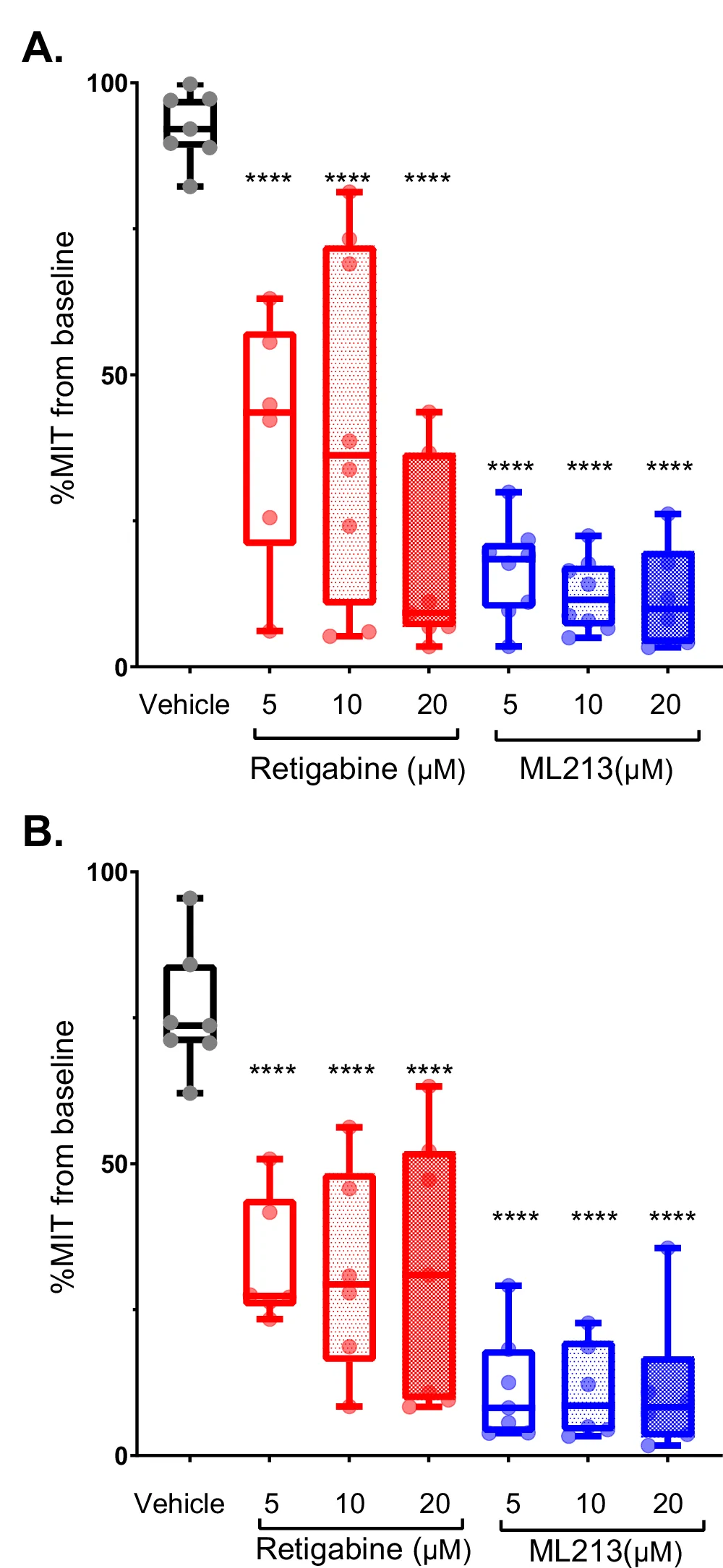
Effects of K_V_7 activators on spontaneous contractility of myometrium from preterm not-in-labor and preterm active-labor mice. Single concentrations of retigabine or ML213 (5, 10 and 20 µM) were added to spontaneously contracting myometrial tissues collected on day 16 post conception from preterm not in labor mice (PTNL; DMSO controls) and preterm active-labor mice (PTAL; RU486-treated). Panels show effects of retigabine and ML213 on mean integral tension (MIT) in PTNL (**A**) and PTAL (**B**) myometrium. MIT was recorded over a 20-minute period after drug or vehicle addition and expressed as a percentage of the receding baseline period. Data are shown as median, interquartile range, and minimum and maximum values. Each concentration was tested using myometrial tissue from individual animals (*n* = 6−8); see Supplementary Data 2 for specific n per group. Statistical significance was determined after testing for normality using one-way ANOVA followed by Holm–Šídák’s multiple-comparisons test. ****P < 0.0001 versus vehicle control.

Our previous work showed the presence of functional K_V_7 channels in non-pregnant and pregnant mouse myometrium, including late pregnancy and LPS-exposed preterm gestations^13^. However, there is limited knowledge of expression or function of myometrial K_V_7 channels related to preterm labor. To rectify this, *Kcnq* and *Kcne* gene expression profiles were assessed in myometrium harvested from a preterm mouse model (RU486 treated on day 15 of gestation to induce progesterone withdrawal and preterm labor and birth on ~day 16) and vehicle (DMSO) treated control mice (Supplementary Fig. 2). Myometrium was obtained from mice when they showed signs of labor (PTAL) and from control non-laboring pregnant mice on day 16 (14 h after DMSO treatment, PTNL) for experiments shown in Figs. 6 and 7. Using myometrium harvested on day 16 of pregnancy, transcripts for all *Kcnq* genes were detected in both PTNL and PTAL myometrium (Fig. 6A, B). Kcnq1, Kcnq4 and Kcnq5 mRNAs were highly expressed.

A separate comparison individual Kcnq mRNA expression data from PTNL versus PTAL myometrium revealed decreases in Kcnq4 (median [IQR]; PTNL, 139.5 [114.6, 259.7]; PTAL, 83.6 [56.7, 106.2], *p* = 0.02) and Kcnq5 (PTNL, 630.8 [443.3, 1025.0]; PTAL, 113.2 [104.6, 351.1], *p* = 0.01) mRNA expression after the onset of preterm labor. Although Kcnq1 showed a reduction in PTAL, this did not reach significance (*p* = 0.065). No differences were observed for Kcnq2 or Kcnq3.

Kcne mRNA expression for isoforms were detected in PTNL (Fig. 6C) and PTAL (Fig. 6D) samples with Kcne4 being the most highly abundant in both groups (*p* = 0.0001 for both).

Comparison of Kcne mRNA expression data from PTNL versus PTAL myometrium showed significant reductions in Kcne2 (median [IQR]; PTNL, 18040 [16183, 226228]; PTAL, 319.3 [231.4, 471.4]; *p* = 0.002) and Kcne1 expression (PTNL, 27.7 [13.1, 35.8]; PTAL, 5.4 [2.8, 8.6]; *p* = 0.02).

### Contribution of K_V_7 channels to the regulation of ex vivo myometrium contractile activity in a mouse model of preterm birth

After characterizing the mRNA expression for *Kcnq* and *Kcne* genes, the functional expression of these channels was assessed using two K_V_7 activators, retigabine and ML213. Figure 7 shows baseline myometrium spontaneous contractility ex vivo in tissues from PTNL and PTL mice (*n* = 6 for each concentration). Application of retigabine to spontaneously contracting myometrium from PTAL mice significantly decreased median MIT at all concentrations tested (Fig. 7B*p* < 0.001). In PTNL samples, similar significant decreases in MIT were only observed (Fig. 7A).

Similar to retigabine, ML213 inhibited contractile activity of PTNL and PTAL myometrium at all concentrations (Fig. 7D, p < 0.001)).

### Repeat dosing of with Kv7 activators delays birth in mouse model of preterm birth

A repeat dosing regimen comprising of up to six gavage treatments (retigabine, ML213, or vehicle) given every 60 min starting ~13 h post RU486 injection was employed.

**Fig. 8:**
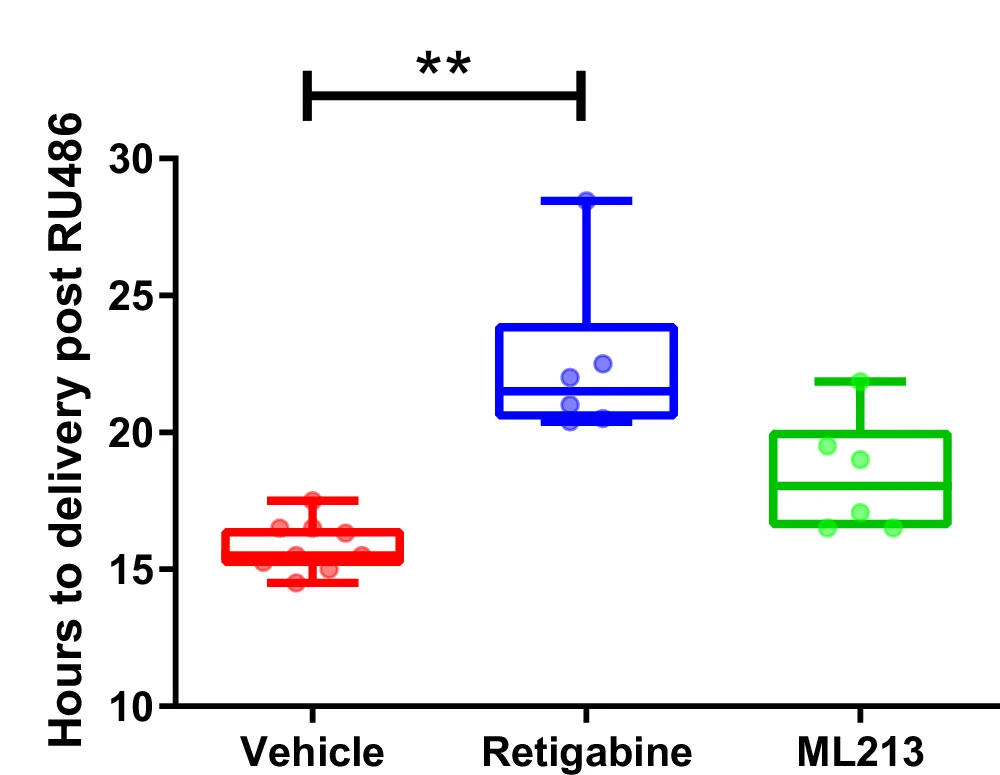
Effect of repeated oral dosing with retigabine or ML213 on time to preterm delivery in RU486-treated mice. Mice received retigabine (20 mg/kg; *n* = 6), ML213 (20 mg/kg; *n* = 6), or vehicle (*n* = 9) by oral gavage beginning 13 h after RU486 administration (150 µg). Doses were given at 60-minute intervals for up to six doses, or until delivery occurred. Mice were continuously monitored by CCTV for signs of labor. Labor onset was defined as delivery of the first pup, and time to delivery was calculated as hours after RU486 administration. Data are expressed as median, interquartile range, and minimum and maximum values. Statistical significance was determined using the Kruskal-Wallis test followed by Dunn’s post hoc multiple-comparison test; **P = 0.003.

Retigabine significantly delayed preterm birth, by increasing the median [IQR] time to delivery of first pup to 21.5 [20.5, 24.0] hours v 15.5 [15.1, 16.5] (*p* < 0.001) hours for RU486 (and vehicle) treated animals; this equated to an average of 39% (36 to 59%) delay in time to delivery (Fig. 8). This is physiologically relevant if compared to the 1 h it takes a mouse to deliver all pups and the ~ 16 h it takes from RU486 administration to induce preterm delivery.

ML213 also delayed delivery (18.0 [16.5,20.1] by ~ 3 h but this was not significantly different from the vehicle control (Fig. 8); this less effective in vivo compared to the tocolytic effect displayed in vitro was anticipated, as it is reported that the drug had poor metabolic stability following incubation with rat or human liver microsomes and was predicted to be rapidly cleared after systemic exposure^36^.

### Fetal viability and fetal ductus arteriosus function

We were unable to assess the impact on neonatal outcomes, as in this PTB model, premature neonates are either born without signs of life/cannibalized by the mother due to their prematurity. This occurred even when gestation was prolonged by K_V_7 activator treatment.

However, in a separate experimental protocol, we assessed the impact of treatment with K_V_7 channel activators on ex vivo murine fetal ductus arteriosus (DA) contractility. This is an important consideration, as agents that constrict the ductus *in utero* would impact fetal perfusion and oxygen delivery^37^. Supplementary Fig. 3 shows that under deoxygenated organ bath conditions that mimic *in utero* oxygen status, fetal ductus arteriosus segments exposed to ML213 had no significant change in lumen diameter (10^-8^ to 10^-4^ M). DAs exposed to retigabine were similarly unaffected over the pharmacologic range (10^-8^ to 10^-6 ^M) although at the highest concentration applied (a concentration unlikely to be used in in vivo), a significant (*p* = 0.0001) constriction was observed at the highest concentration (10^-4^M) compared to baseline diameter. Because of this, there was a significant (*p* = 0.0001) difference in E_max_-values between these two drugs.

## Discussion

Ion channels represent an attractive class of drug targets owing to their central involvement in cellular physiology and disease, accounting for an estimated 10–20% of small-molecule therapeutic targets^38,39^. Within this group, K_V_ channels form a large gene and well-characterized superfamily that has been studied extensively in smooth muscles, including the myometrium, in terms of function and therapeutically^9,40–47^. In particular, the clinical development of K_V_7 channel modulators has progressed clinically for disorders such as epilepsy, hypertension, stroke, lung disease, overactive bladder, and neuropathic pain^41,48–54^, highlighting the clinical tractability of this channel family. Against this background, the present work suggests that modulation of uterine excitability, through K_V_7.2–7.5 channels, can influence the timing of parturition. The accompanying molecular and functional data from human pregnant myometrium support the relevance of this mechanism across species.

Beyond our previous work^12–14^ and available RNA-seq datasets^55^, relatively little is known about the functional role of K_V_7 channels in the laboring human myometrium. In general, K channel activity contributes to suppression of uterine contractility in pregnancy through promoting a low resting potential, modifying action potentials through repolarization, and hence limiting calcium entry and force production^12^. However, as gestation nears term and labor (in both rodents and humans), there is a transition to greater myometrial cell excitability due to a gradual increase of the resting membrane potential, most likely due to change in K^+^ channel populations and activity^56,57^. For a K^+^ channel to be useful for inhibiting preterm labor, its expression must be maintained in labor. K_V_11.1/hERG channels, for example, are expressed in late pregnancy, but their ability to enhance repolarization is suppressed at labor onset through a variety of pathways^12,45,58,59^. Our previous pilot work in human myometrium suggested that K_V_7.2-5 were expressed in human myometrium in late pregnancy, and here we further confirm through molecular profiling that KCNQ and KCNE mRNA are expressed both at the end of pregnancy and after the onset of labor. We also present initial evidence for K_V_7.4 and KCNE4 RNA expression in the myometrium in tissues from pregnancies at the time of spontaneous preterm labor, albeit the sample size is low and requires further validation. Of the different isoforms found, KCNQ4 and KCNE4 mRNAs were the most highly expressed transcripts in terms of labor and were also expressed in the myometrium from pregnancies at the time of preterm labor. This was reflected in K_V_7.4 and KCNE4 protein expression in the myometrium from term and in labor pregnancies. This identifies K_V_7.4 as a potential K_V_7 channel target in human myometrium^37^. Whilst transcripts for KCNQ5 were found and maintained in labor, we did not detect K_V_7.5 protein. This does not fully rule out this as a target but more work on understanding protein expression is needed. KCNQ3 whilst expressed, is a key component of the neuronal M channel, so a less favorable target^60^ along with Kv7.1, which is a cardiac-associated K_V_ channel.

Pharmacological approaches using retigabine and ML213 provide clear evidence that activation of K_V_7.2-5 channels can suppress contractions in human myometrium in vitro, an observation similar to that in term pregnant mice^13^. ML213 is reported as a K_V_7.4, K_V_7.2, and K_V_7.5 channel activator, but given that K_V_7.2 was expressed at low levels in the myometrium, these findings support the notion that K_V_7.4 channels are a plausible functional human myometrial target.

The K_V_7 channel inhibitor, XE991, also altered human uterine contractility in vitro, providing additional evidence for functional K_V_7. In contrast, although KCNQ1 transcripts (K_V_7.1) were detectable in human myometrium, K_V_7.1 did not appear to contribute to contractile function, as chromanol application had no effect.

Two options for inducing PTB were considered: a lipopolysaccharide-induced PTB (inflammation-driven) and RU486 (progesterone/glucocorticoid block). The latter was chosen because it may be more reflective of idiopathic PTB, a clinical scenario that would benefit from an effective tocolytic. RU486 induction of PTB is rapid in the mouse due to dependence on progesterone for maintenance of pregnancy; therefore, even a modest delay in labor in this context is supportive of a meaningful proof-of-principle effect.

The effectiveness of Kv7.2-5 activation to prevent preterm labor was demonstrated in a mouse model where K_V_7 channels components, particularly *kcnq4 and kcnq5*, were highly expressed in the myometrium of preterm mice (prior to and after the onset of labor). Both retigabine and ML213 suppressed contractility in tissue ex vivo from preterm mice (not in labor and in labor).

This effect was also confirmed in vivo where repeated gavage of retigabine significantly delayed labor and birth of the first pup by six hours compared to controls.

ML213 was also assessed, although we were aware prior to starting experimentation that it would quickly be metabolized by the liver when given orally. With this caveat, as a K_V_7.4 (and Kv7.4/5, Kv7.5) targeting agent, it showed significant promise as it delayed preterm birth by three hours. Focusing on the development of more favorable administration routes for an ML213 drug may be preferable.

In the context of human pregnancy, findings from this animal model suggest that K_V_7.2–K_V_7.5 activators could potentially delay preterm birth for at least a week. In clinical terms, even short-term prolongation of pregnancy can be meaningful if it permits corticosteroid administration, magnesium sulfate for neuroprotection and transfer to an appropriate neonatal care setting. This timeframe also aligns with the standard endpoint used in clinical trials evaluating tocolytic efficacy, making it both clinically and scientifically relevant^61,62^.

In terms of limitations, there are several avenues available for further exploration. There is a need for further interrogation of the cellular location and the composition of the K_V_7.4 channels (homo or heterotetramers with other KCNQ isoforms such as KCNQ5)^63,64^, or KCNE accessory subunits using proximity assays, imaging, patch clamp electrophysiology, and transgenic mouse models, and through increasing numbers of human samples from preterm labor studied.

In the current study, we restricted functional organ bath studies to myometrium obtained from pregnancies in late pregnancy, as samples from pregnancies at the time of spontaneous preterm labor are rare, with biopsies often too small for organ bath studies and taken out of working hours. However, with time, these experiments could be collated. While the initiating pathways for preterm labor may differ from those at term, the core mechanisms underlying myometrial contractility are likely to be conserved. This supports the use of term tissues (both prior to labor onset and in labor) as a practical compromise for essential early-phase mechanistic work. This approach allows determination of effective compound concentrations and pathway responses. Going forward, these findings can guide and prioritize essential validation experiments using valuable but scarce preterm tissues. In addition, term tissues exposed to inflammatory mediators could, in the future, be developed to better model infection-associated preterm labor. In vivo studies using a mouse model of PTB were also undertaken to address this limitation of human tissue availability and illustrated the potential utility of K_V_7 channel modulation and provided an in vivo framework to support the findings from human tissue studies.

Uterine contraction at the tissue level is similar between species, and tissues exhibited relevant KCNQ and KCNE expression profiles. However, before work can be translated into human pregnancy, this may necessitate additional studies in an alternative animal model that does not rely on uterine peristalsis for pup delivery and is suitable for study of longer-term impacts on neonates, particularly if more specific K_V_7.4 activators are to be developed. The side effects on other tissues, in particular cardiovascular and nervous systems, and smooth muscles^10,65^, which also express K_V_7.2-5 would also have to be monitored, although in the mouse model, retigabine was well tolerated.

There is still a need to assess the impact of K_V_7 activators on neonatal outcome. In our study, neonatal wellbeing could not be reviewed due to the gestation-based immaturity of the mice in this model, even when gestation was prolonged. However, we considered the impact of retigabine and ML213 ex vivo on the murine fetal ductus arteriosus, and whilst retigabine at the highest concentration caused some constriction, this was at concentrations not likely to be achieved in vivo in mice. In future studies, we plan to assess compounds in terms of pregnancy or a modified PTB model (induced later in gestation or with a milder stimulus) where neonates survive to determine neonatal developmental and behavioral outcomes. To support translation of these findings into K_V_7.4 (or K_V_7.4/5) based tocolytics, protocols investigating different routes and drug delivery mechanisms may be useful.

In summary, these data support K_V_7.2-5 activators as emerging candidates as a tractable strategy for drug development. These activators can clearly suppress uterine contractility in human tissues and delay PTB in mice. There is compelling evidence that K_V_7.4 and associated accessory subunit KCNE4 are the targets for uterine-specific drug development^11^. Pharmaceutical companies have historically invested relatively little in maternity-focused therapeutics. However, with the continued increase in PTB and a clearer understanding that the burden of prematurity is greatest among low-risk pregnancies, there is a pressing need for renewed strategic investment and research in this field^5^.

While opportunities to explore drug repurposing have been affected by concerns about the long-term effects of retigabine and flupirtine^65,66^, release of new or existing K_V_7 targeting compounds from drug libraries^23,34,52^ could potentially facilitate research and drug development for the more short-term use needed in this major area of health care. This could be underpinned by focusing on developing more precise uterine targeting (to avoid off target cardiovascular effects), using emerging drug delivery systems^53,54^.

## Supplementary information

Supplementary Information

Description of Additional Supplementary files

Supplementary Data 1

Supplementary Data 2

## Data Availability

No data were generated that require mandatory public repository, but individual numerical data for graphs has been provided in the Supplementary Data 2 file with an individual excel sheet per figure. Limited residual human myometrial tissue and associated clinical data are available under controlled access only. Samples are finite and their use is restricted by the conditions specified in the study participant information sheet and consent form. Associated personal clinical information is governed by GDPR requirements and will require full anonymisation. Any proposed use outside of the original consent would require appropriate ethical approval and potentially participant re-consent. Requests from bona fide researchers should be sent to Professor Rachel Tribe at rachel.tribe@kcl.ac.uk and they will be asked to complete a sample and data request form including a project summary, description of experimental design and statistical analysis plan, intended use, ethical approvals and governance arrangements. Requests will normally be reviewed within 4–8 weeks. Approved access will be subject to tissue availability, confirmation that the proposed use is consistent with participant consent and ethical approvals, and completion of appropriate data/material transfer agreements with King’s College London and Guy’s and St Thomas’ NHS Foundation Trust. Agreements will restrict use to the approved project and prohibit re-identification, onward sharing or commercial use without further permission. Uncropped western blots are provided at the end of the Supplementary Information document. The source data for all primers and antibodies used are in Supplementary Data File 1. The source data for other figures (where relevant) are in Supplementary Data File 2
