# Supplementary material for: KV7 channel activation inhibits human and murine myometrium contractility and delays delivery in a mouse model of preterm birth
Source: Commun Med (Lond). 2026 Sep 3;6:447. doi: 10.1038/s43856-026-01861-7 (PMC13542272; doi:10.1038/s43856-026-01861-7)

Title: K_V7 channel activation inhibits human and murine myometrium contractility and delays delivery in a mouse model of preterm birth

Yosef T. Mansour[1†], Hiten D. Mistry[1,2†], Evonne C. Chin-Smith[1], Mariola K. Zaleska[1], Deanna C. Sekulich[3], Jennifer L. Herington[3], Jeff Reese[3], Rima Patel[1], Philip I. Aaronson[4], Donna M. Slater[5], Sarah K. England[6], Iain A. Greenwood[7], Mark R. Johnson[8], Paul D. Taylor[1], Rachel M. Tribe[1*]

Affiliations:

[1]Department of Women and Children's Health, School of Life Course and Population Sciences, Faculty of Life Sciences and Medicine, King's College London; London SE1 7EH, UK

[2]Division of Public Health & Epidemiology, College of Life Sciences, University of Leicester, LE1 7RH, UK

[3]Department of Pediatrics, Vanderbilt University Medical Centre, Nashville, TN, USA

[4]Department of Inflammation Biology, School of Immunobiology and Microbial Science, Faculty of Life Sciences and Medicine, King's College London; London SE1 9RT, UK

[5]Department of Physiology and Pharmacology and Dept. of Obstetrics and Gynecology, Cumming School of Medicine, University of Calgary, Calgary; Alberta, Canada

[6]Center for Reproductive Health Sciences, Department of Obstetrics and Gynecology, WashU Medicine; St. Louis, MO, USA

[7]School of Health & Medical Sciences, City St George's University of London; London UK

[8]Faculty of Medicine, Department of Surgery & Cancer, Imperial College London; London UK

[†]These authors have contributed equally to this work

*Corresponding author: rachel.tribe@kcl.ac.uk

SUPPLEMENTARY INFORMATION

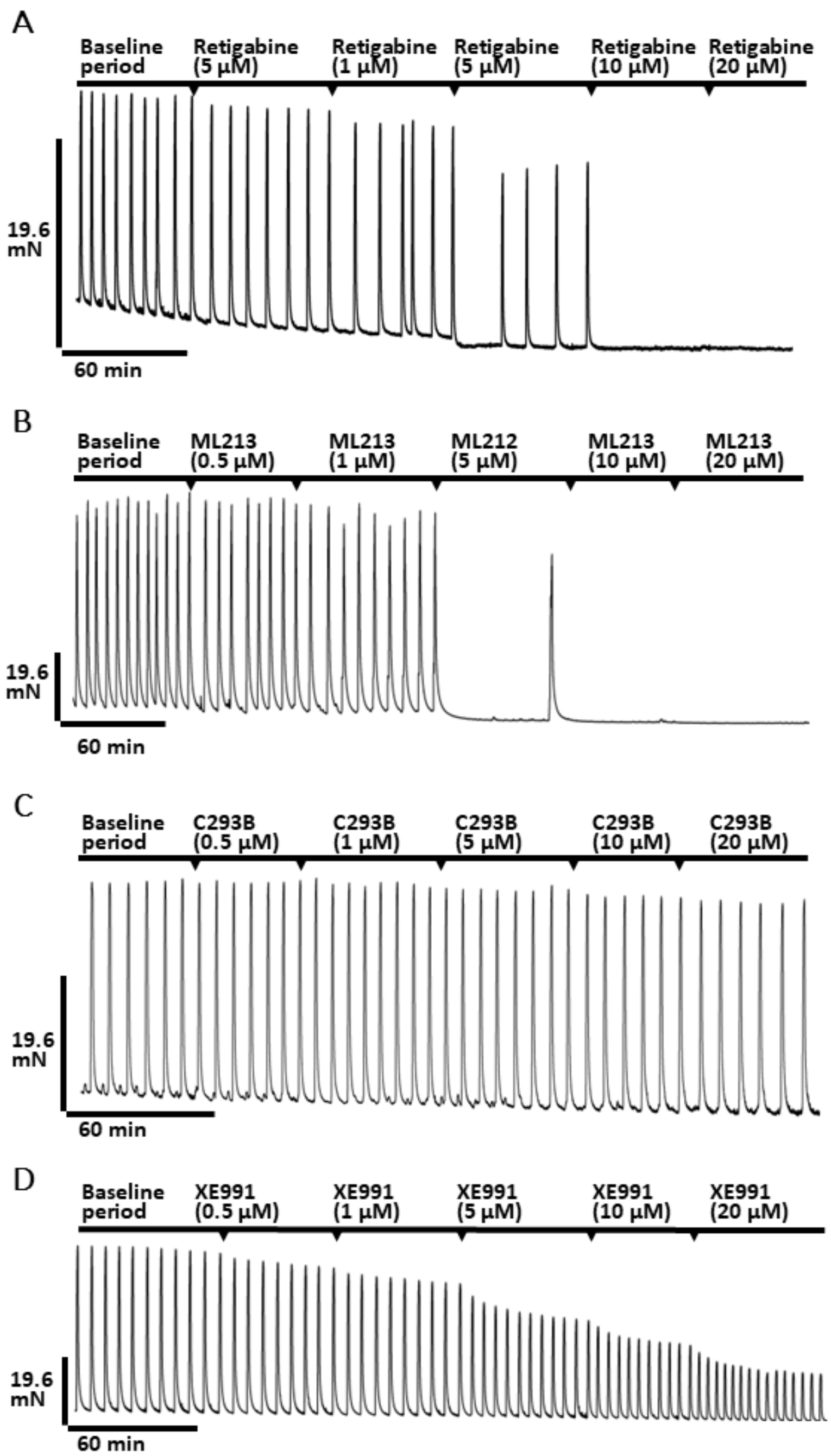

Supplementary Figure 1. Effect of K_V7 channel modulators on spontaneous contractility of human myometrium. Representative traces show the cumulative dose effect (0.5 to 20 µM) of the $K_V7.2$–5 activators retigabine (A) and ML213 (B), chromanol, a $K_V7.1$ inhibitor, and the pan-K_V7 inhibitor XE991 on spontaneous contractility of ex vivo myometrium tissue segments taken from term pregnancies prior to labor onset (TNL).

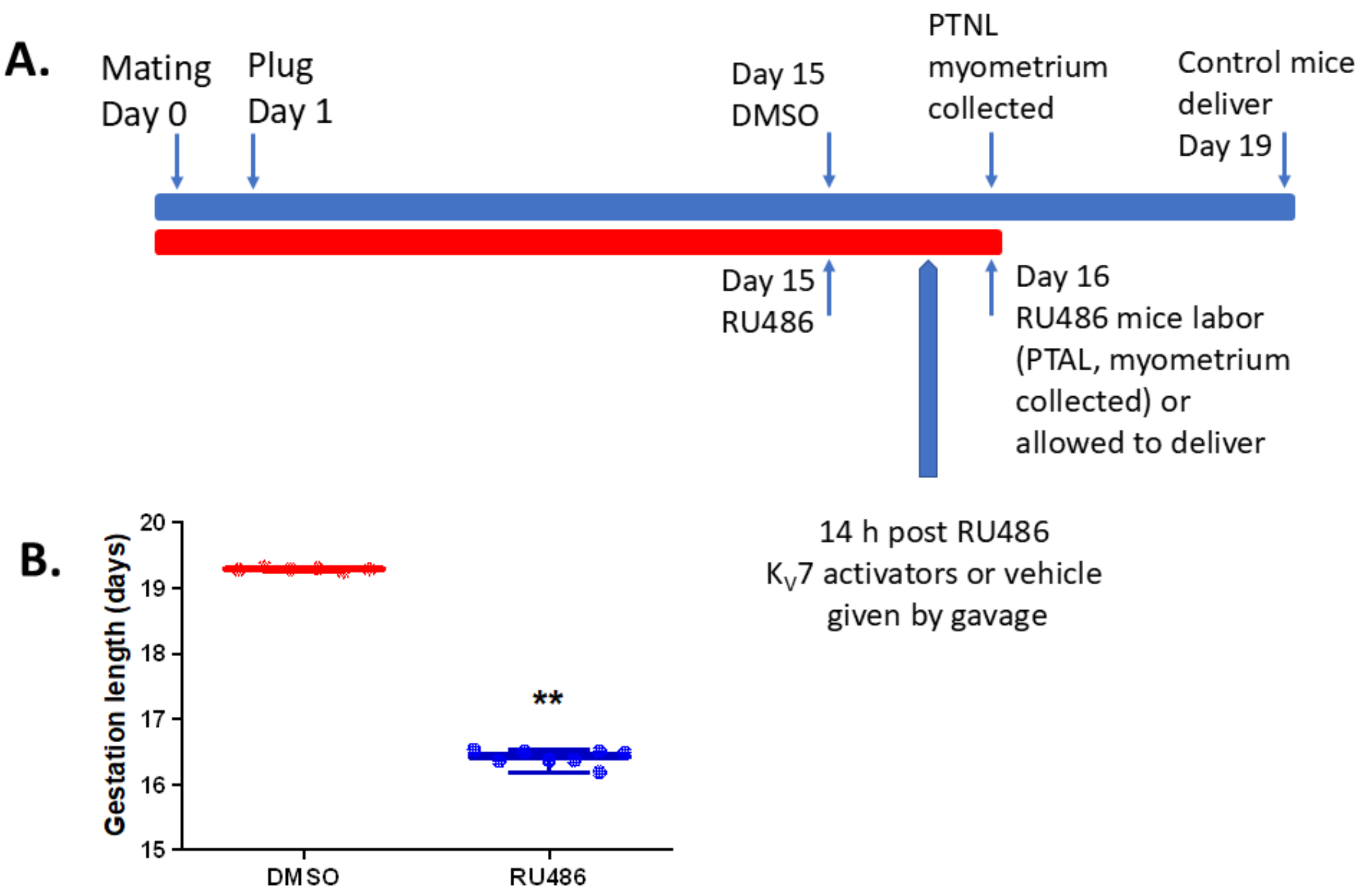

Supplementary Figure 2. Experimental protocol for assessing impact of K_V7 activators on preterm birth in mice. (**A**) **S**chematic of the experimental protocol used to induce preterm labor and birth in pregnant C57BL/6J mice. Mice were mated (day 0) with plug confirmed on day1 followed by daily weighing up to days 8-10; female mice were approximately 13 weeks old. On day 15, mice were injected intraperitonially with either RU486 (150 µg dissolved in DMSO at a concentration of 1 µg/ml) or DMSO (150 µl) alone. For organ bath experiments, animals were sacrificed, and myometrium was collected on day 16 from DMSO controls (preterm not in labor, PNL) and RU486 treated animals at the time of delivery (preterm in labor, PTAL). Other mice were used for timing of gestation length and assessment of the effect of K_V7 activator treatments (see methods for details) given by gavage. (**B**) Effect of RU486 administration on gestation length. Using CCTV, the delivery of the first pup defined end of gestation. RU486 treated mice (n=6) consistently delivered on day 16, control animals (n=8) on day 19 (**p<0.0003). Data are expressed as median, interquartile ranges and minimum and maximum values analyzed with two-tailed Mann-Whitney test.

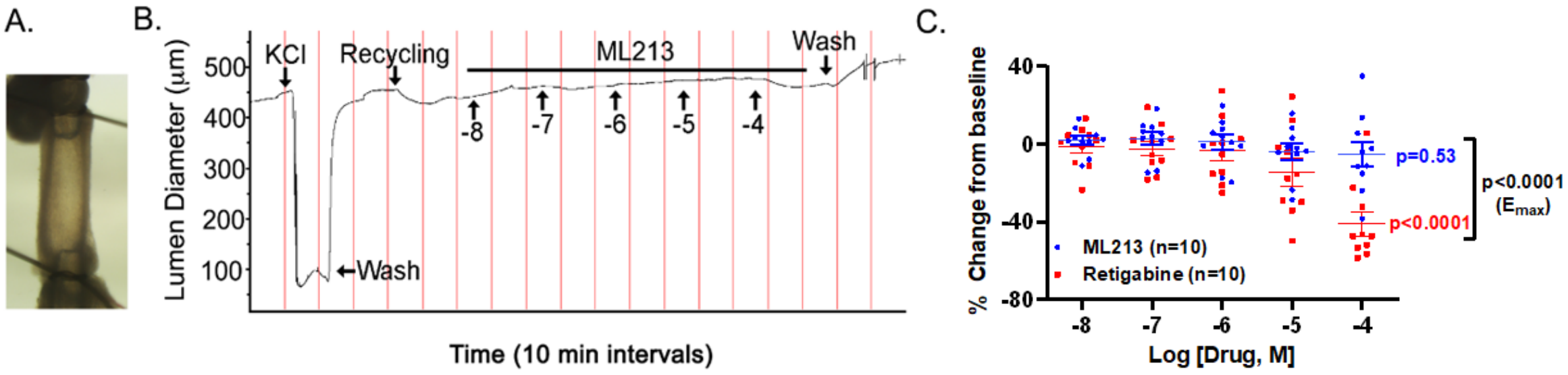

Supplementary Figure 3. Effect of K_V7 channel activation on fetal ductus arteriosus (DA) tone. (**A**). Isolated DAs from term gestation fetal mice were mounted on glass pipettes for pressure myography. (**B**). Representative tracing of an isolated fetal DA in response to 50 mM KCl to verify vessel reactivity, followed by exposure to increasing concentrations of ML213 (10^{-4} to 10^{-8} M). (**C**). Cumulative concentration-response curves show that exposure to increasing concentrations of ML213 had no effect on DA diameter under fetal oxygen conditions compared to baseline (p=0.53). Constriction of the isolated DA was noted after exposure to the highest concentrations of retigabine (p<0.0001) compared to baseline diameter. The Emax of retigabine versus ML213 was significantly different (P <0.0001). Data are presented as mean ± S.E.M., representing DA tissues from 5 different litters, n=10 DA tissues for ML213 and n=10 DA tissues for retigabine.

Uncropped Western Blots used for the data provided in Figure 4A.

1. For Kv7.1

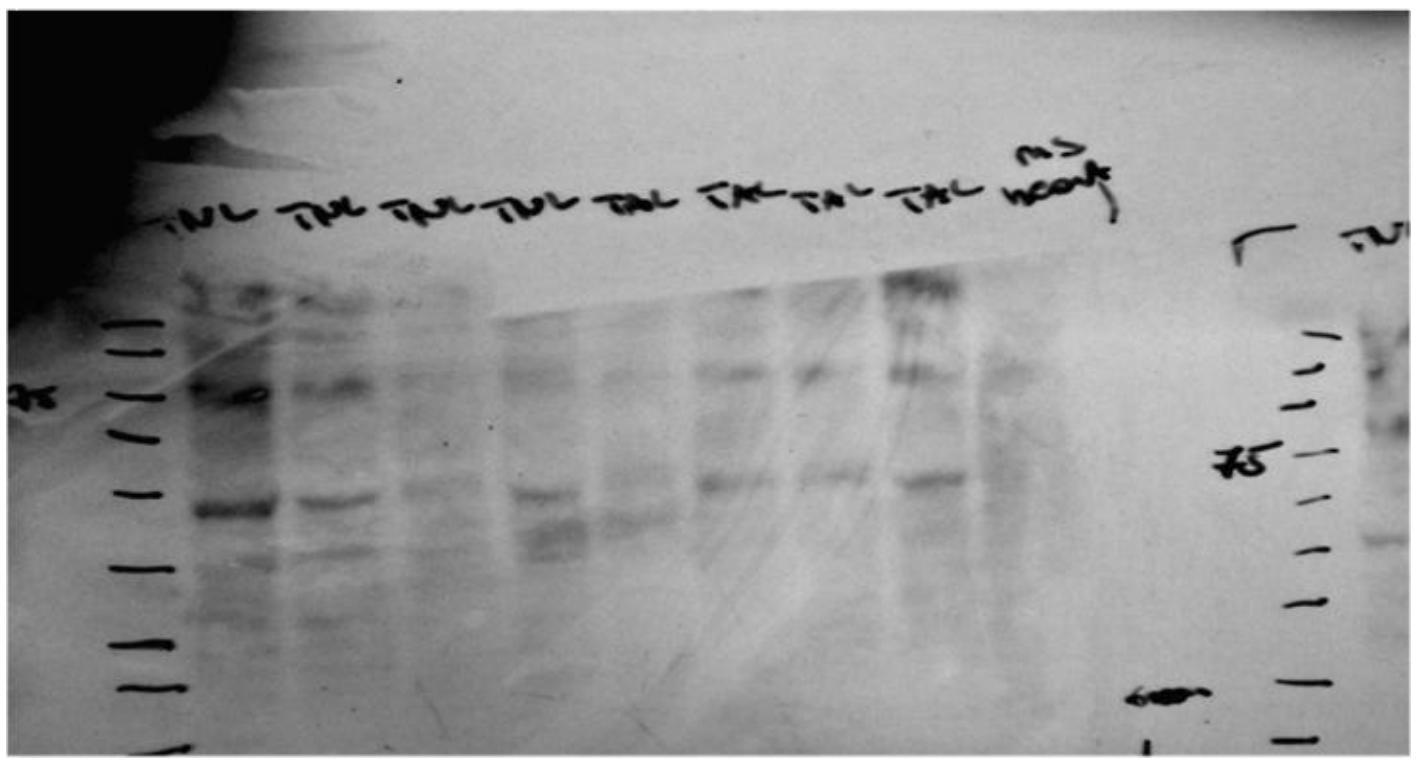

2. For Kv7.4

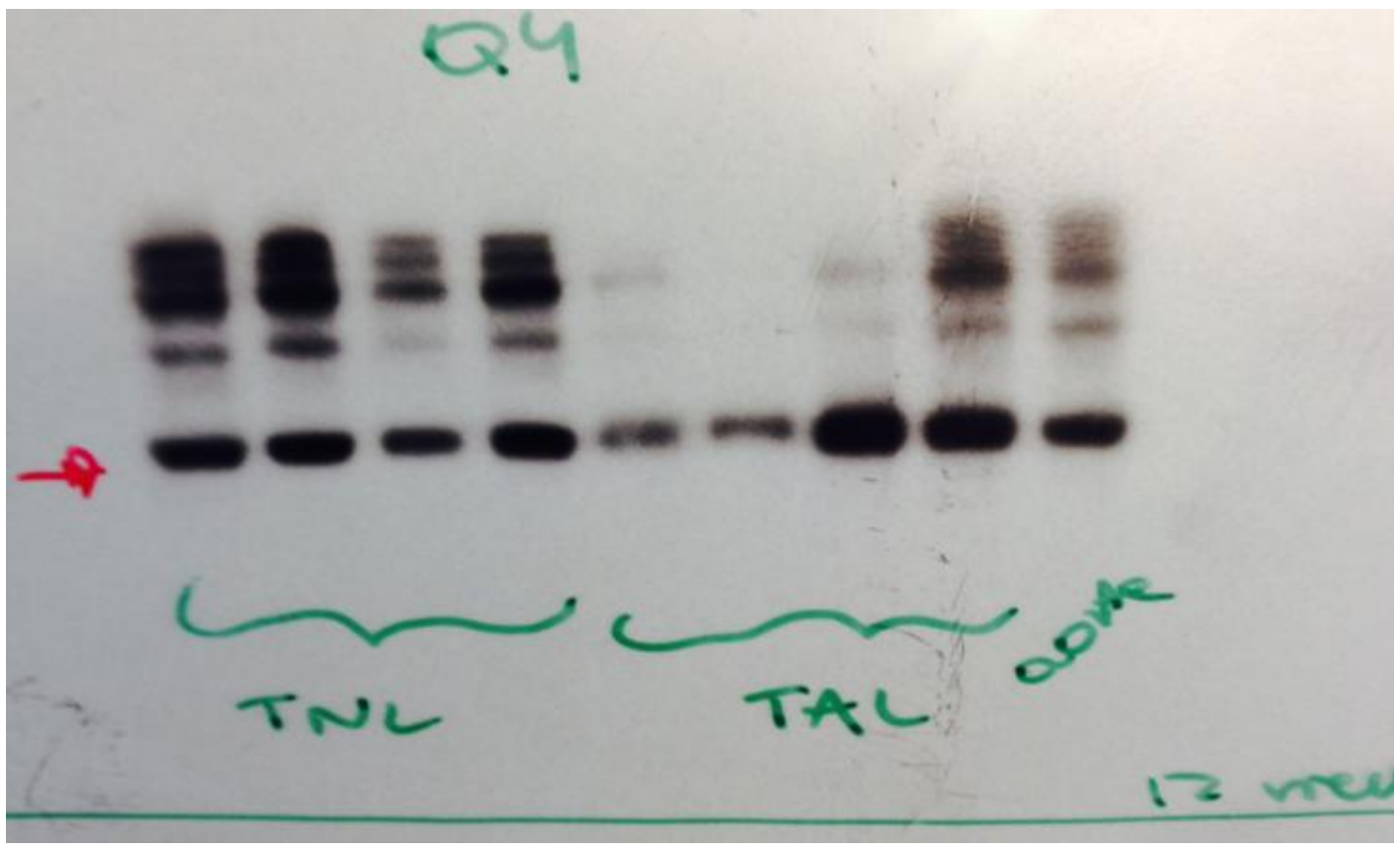

3. For KCNE2_1

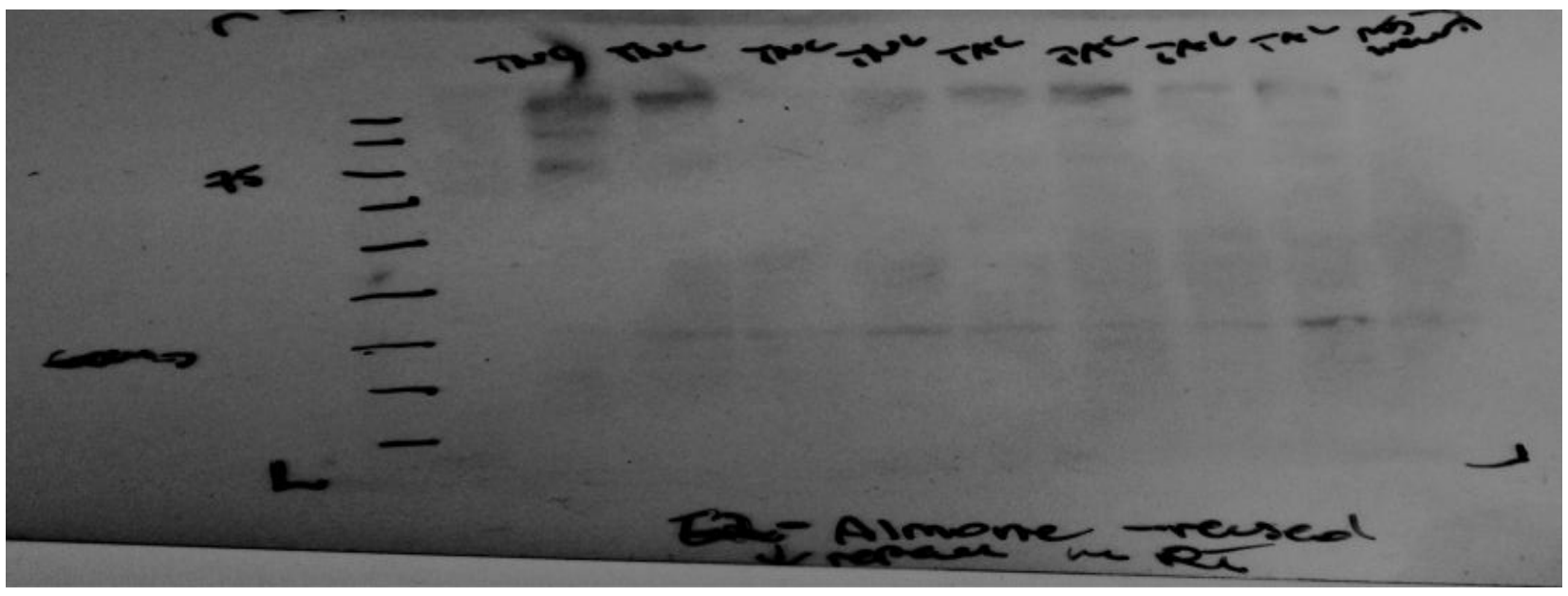

4. For KCNE2_2

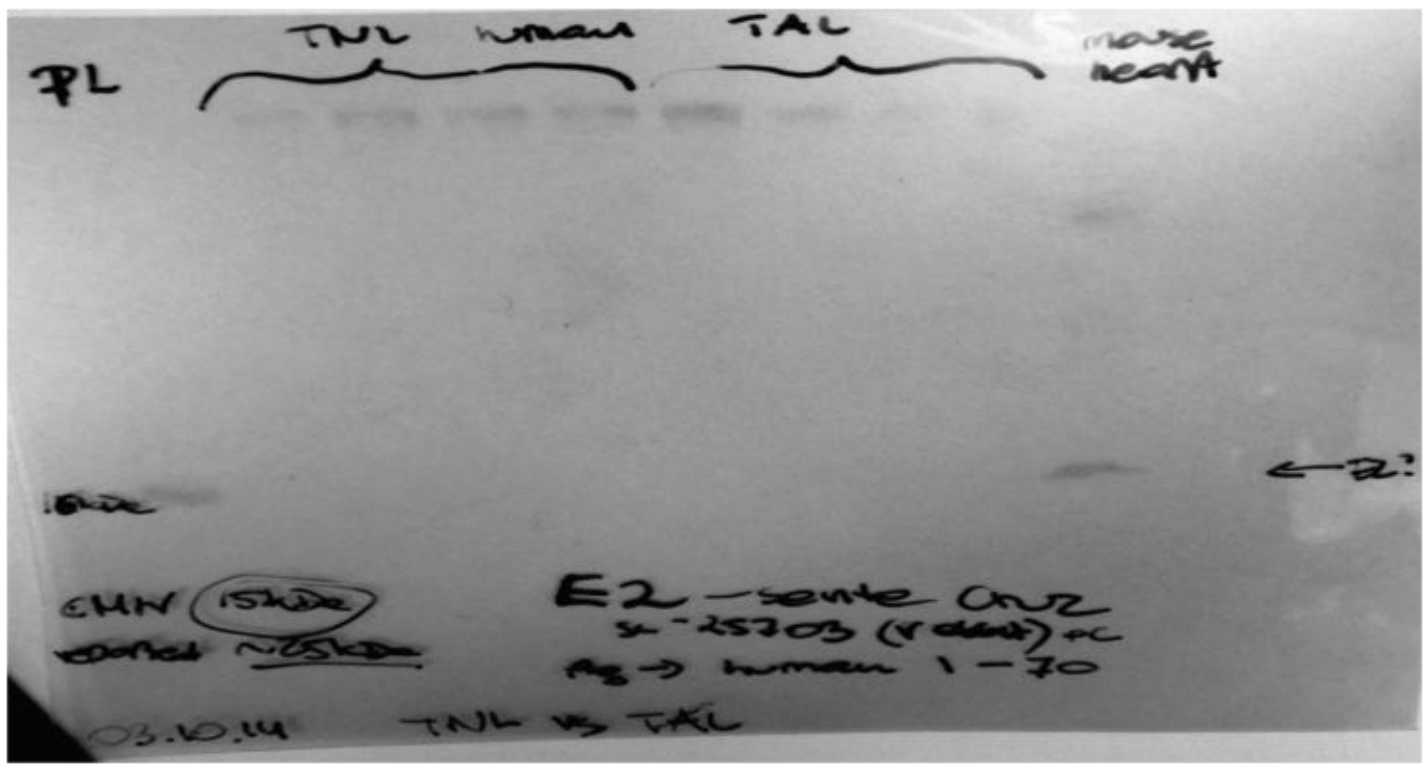

5. For KCEN4

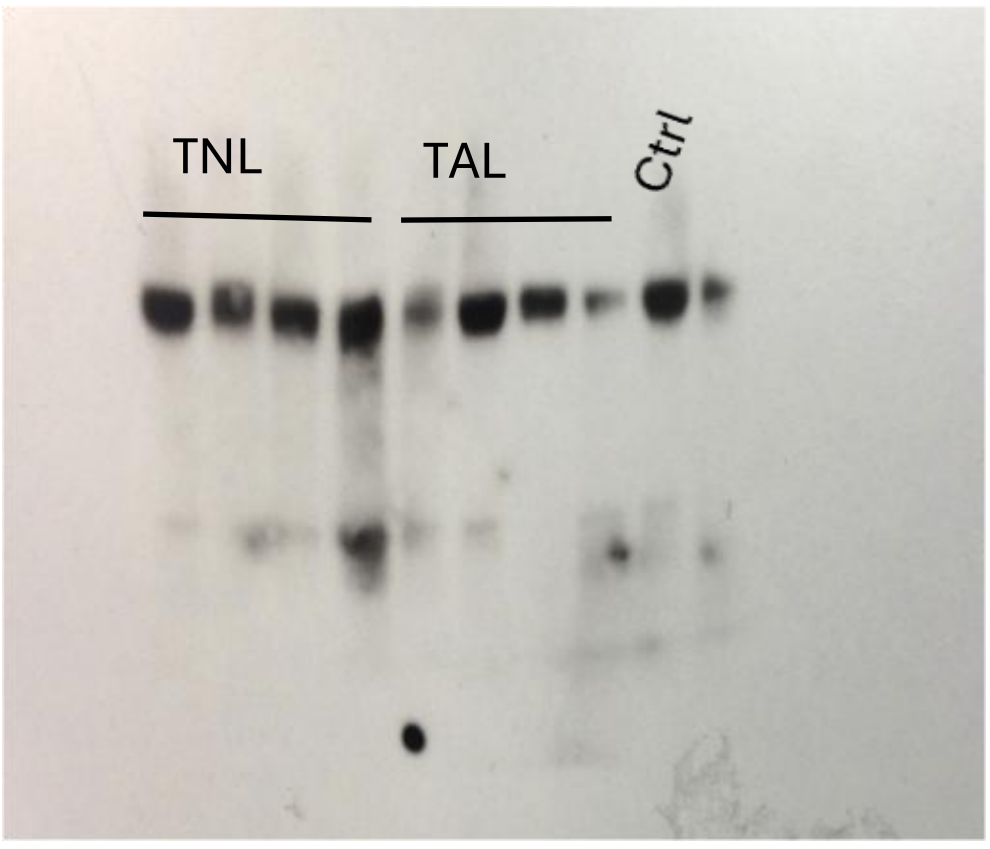

6. For Smooth muscle alpha actin (SMAA)

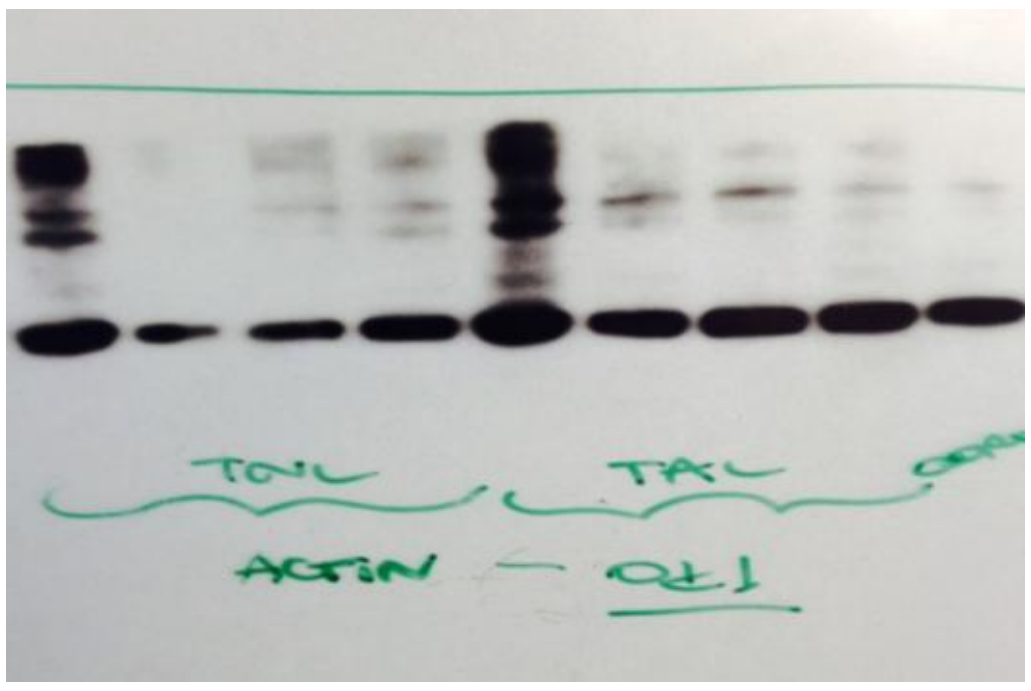

Supplement: Supplementary file 1 [file 43856_2026_1861_MOESM1_ESM.pdf]
