# Supplementary material for: KV7 channel activation inhibits human and murine myometrium contractility and delays delivery in a mouse model of preterm birth
Source: Commun Med (Lond). 2026 Sep 3;6:447. doi: 10.1038/s43856-026-01861-7 (PMC13542272; doi:10.1038/s43856-026-01861-7)
Supplement: Supplementary file 2 [file 43856_2026_1861_MOESM2_ESM.docx]

**Description of Additional Supplementary Files**

File name: Supplementary Data 1

Description: Supplementary tables 1-4

File name: Supplementary Data 2

Description: Data for Figures 1-8 and Supplementary Figures 2B and 3C
